# CDR3α drives selection of the immunodominant Epstein Barr virus (EBV) BRLF1-specific CD8 T cell receptor repertoire in primary infection

**DOI:** 10.1371/journal.ppat.1008122

**Published:** 2019-11-25

**Authors:** Larisa Kamga, Anna Gil, Inyoung Song, Robin Brody, Dario Ghersi, Nuray Aslan, Lawrence J. Stern, Liisa K. Selin, Katherine Luzuriaga

**Affiliations:** 1 Program in Molecular Medicine, University of Massachusetts Medical School, Worcester, Massachusetts, United States of America; 2 Department of Pathology, University of Massachusetts Medical School, Worcester, Massachusetts, United States of America; 3 School of Interdisciplinary Informatics, University of Nebraska at Omaha, Nebraska, United States of America; National Cancer Institute, UNITED STATES

## Abstract

The T cell receptor (TCR) repertoire is an essential component of the CD8 T-cell immune response. Here, we seek to investigate factors that drive selection of TCR repertoires specific to the HLA-A2-restricted immunodominant epitope BRLF1_109-117_ (YVLDHLIVV) over the course of primary Epstein Barr virus (EBV) infection. Using single-cell paired TCRαβ sequencing of tetramer sorted CD8 T cells *ex vivo*, we show at the clonal level that recognition of the HLA-A2-restricted BRLF1 (YVL-BR, BRLF-1_109_) epitope is mainly driven by the TCRα chain. For the first time, we identify a CDR3α (complementarity determining region 3 α) motif, KDTDKL, resulting from an obligate AV8.1-AJ34 pairing that was shared by all four individuals studied. This observation coupled with the fact that this public AV8.1-KDTDKL-AJ34 TCR pairs with multiple different TCRβ chains within the same donor (median 4; range: 1–9), suggests that there are some unique structural features of the interaction between the YVL-BR/MHC and the AV8.1-KDTDKL-AJ34 TCR that leads to this high level of selection. Newly developed TCR motif algorithms identified a lysine at position 1 of the CDR3α motif that is highly conserved and likely important for antigen recognition. Crystal structure analysis of the YVL-BR/HLA-A2 complex revealed that the MHC-bound peptide bulges at position 4, exposing a negatively charged aspartic acid that may interact with the positively charged lysine of CDR3α. TCR cloning and site-directed mutagenesis of the CDR3α lysine ablated YVL-BR-tetramer staining and substantially reduced CD69 upregulation on TCR mutant-transduced cells following antigen-specific stimulation. Reduced activation of T cells expressing this CDR3 motif was also observed following exposure to mutated (D4A) peptide. In summary, we show that a highly public TCR repertoire to an immunodominant epitope of a common human virus is almost completely selected on the basis of CDR3α and provide a likely structural basis for the selection. These studies emphasize the importance of examining TCRα, as well as TCRβ, in understanding the CD8 T cell receptor repertoire.

## Author summary

EBV is a ubiquitous human virus that has been linked to several diseases, including cancers and post-transplant lymphoproliferative disorders. CD8 T cells are important for controlling EBV replication. Generation and maintenance of virus-specific CD8 T cells is dependent on specific interaction between MHC-peptide complexes on the infected cell and the TCR. In this study, we performed single cell sequencing of paired TCR α and β chains from EBV-specific CD8 T cells isolated at two time points (primary infection and convalescence) from four individuals undergoing acute EBV infection. We describe a TCRα sequence that was shared by all four individuals and identify conserved residues within this sequence that likely contribute to viral recognition. Examination of the crystal structure of the peptide-MHC complex and subsequent experimental data suggest that a specific interaction between a negatively charged aspartic acid at position 4 of the peptide and a positively charged lysine in the TCR may be particularly important. These findings are highly relevant to current efforts to understand how the TCR repertoire may contribute to or protect against disease, the development of TCR diagnostics for diseases, and at improving the efficacy of T cell based therapies.

## Introduction

EBV infects almost 95 percent of the world’s population by the fourth decade of life. In older children and adults, primary infection with EBV often manifests as acute infectious mononucleosis (AIM), a self-contained illness characterized by fever, pharyngitis, lymphadenopathy and malaise. Activated virus-specific CD8 T cells are commonly expanded in the peripheral blood in AIM followed by a contraction in convalescence. EBV-specific memory CD8 T cells provide protection from EBV reactivation [1–5]. In HLA-A:02+ individuals, the EBV-specific CD8 T-cell response is dominated by responses to two lytic cycle proteins, BRLF1 (BRLF1_109-117_ epitope: YVLDHLIVV) and BMLF1 (BMLF1_280-288_ epitope: GLCTLVAML) [6, 7].

The interaction of CD8 TCRs with virus-derived peptides bound to MHC-I molecules (pMHC) expressed on an infected cell surface [8–11] confers specificity of the CD8 T-cell response. The TCR repertoire is an important determinant of CD8 T-cell-mediated antiviral efficacy or immune-mediated pathology [12–17]. Each TCR is a membrane-bound, heterodimeric protein that is formed from two polypeptides: α and β. TCR diversity is generated through recombination events, in which each chain arises from a random rearrangement of variable (V), diversity (D), joining (J) and constant (C) gene segments [18]. The TCRβ chain rearranges first, followed by the TCRα; the TCRα chain keeps rearranging until a TCRα chain has been rearranged that is capable of successfully pairing with the TCRβ chain. The TCR is then expressed on the surface of the T cell. Due to this sequence of events it is not unusual to find T cells with the same TCRβ chain paired with different TCRα; reports of the reverse are, however, rare. This recombination process results in a diverse pool of unique TCRα and β clonotypes. Additions of non-templated (N) nucleotides or deletions of nucleotides at the V(D)J junctions, commonly known as the complementarity-determining region 3 (CDR3) and pairing of different TCRα and β segments further enhance the overall diversity of the TCR repertoire, estimated to range from 10^15^−10^20^ unique potential TCRαβ clonotypes [15, 19].

TCR repertoire analyses enable us to visualize the clonotypic identity of TCRs and to glean information about important features that dictate antigenic specificity or recognition [20, 21]. Novel sequencing methods have shed important insights into the composition and organization of the TCR repertoires of common pathogens. Despite the multitude of V and J genes and virtually limitless number of TCRs that can be made from V(D)J recombination, skewing of pathogen-specific repertoires have been observed [22–26]. These skewed repertoires have been observed in the forms of preferential usage of particular V and/or J genes, as well as in conservation of CDR3 motifs or sequences within and across individuals. There has been a growing interest in understanding how these biases emerge, their immunological relevance, and their implications for either protection or immunopathology [12, 20, 21, 26–28]. A caveat of the majority of studies to date is that they have focused primarily on analyses of the TCRβ chain, with little regard for the TCRα chain. One of the reasons that the mechanisms that shape T-cell memory through TCRα selection have been difficult to delineate is due to technical constraints associated with the lack of VA-family specific antibodies. A T cell’s ability to co-express two α-chains may also contribute [29, 30]. We performed single-cell TCRαβ sequencing of immunodominant GLC-BM (BMLF-1_280_ epitope) and YVL-BR-specific CD8 T cells directly ex vivo over the course of primary infection and applied a newly developed analytical tool [20] for the identification of significantly enriched features in epitope-specific TCR repertoires. This revealed selective use of particular AV genes in the YVL-specific TCR repertoire, as well as identified novel pairing relationships between the α and β TCR chains. We identified a TCRα chain, AV8.1-KDTDKL-AJ34, which was shared by all study participants. Additionally, this chain was degenerate and paired with multiple different TCRβ chains in the same individual, suggesting that it might be important for antigen recognition. To gain insight into a potential basis for the conserved use of this TCRα chain, we solved the crystal structure of the YVL-BR peptide-bound and uncovered a solvent-exposed Asp(D) at position 4 of the peptide. Further examination of potential structural constraints suggests that a conserved Lys(K) within the CDR3α of the TCRα clones may mediate contact with the protruding Asp(D) on the YVL-BR peptide-bound MHC.

## Results

### Patient characteristics

Four HLA-A*02:01+ individuals presenting with symptoms of AIM and laboratory studies consistent with primary infection were studied (**S1 Table**) at initial clinical presentation (AIM) and 5–8 months later (Convalescence; CONV). Direct tetramer staining of peripheral blood revealed that 2.7%±0.7 (mean±SEM) and 1.3%±0.3 of CD8 T cells were YVL-BR- and GLC-BM-specific, respectively, in AIM; the frequencies of YVL-BR- and GLC-BM-specific CD8 T cells declined to 0.3%±0.7 and 0.3%±0.1, respectively, in CONV.

### The TCR repertoire is individualized and qualitative features distinguish YVL-BR- and GLC-BM-specific TCR repertoires

**Fig 1:**
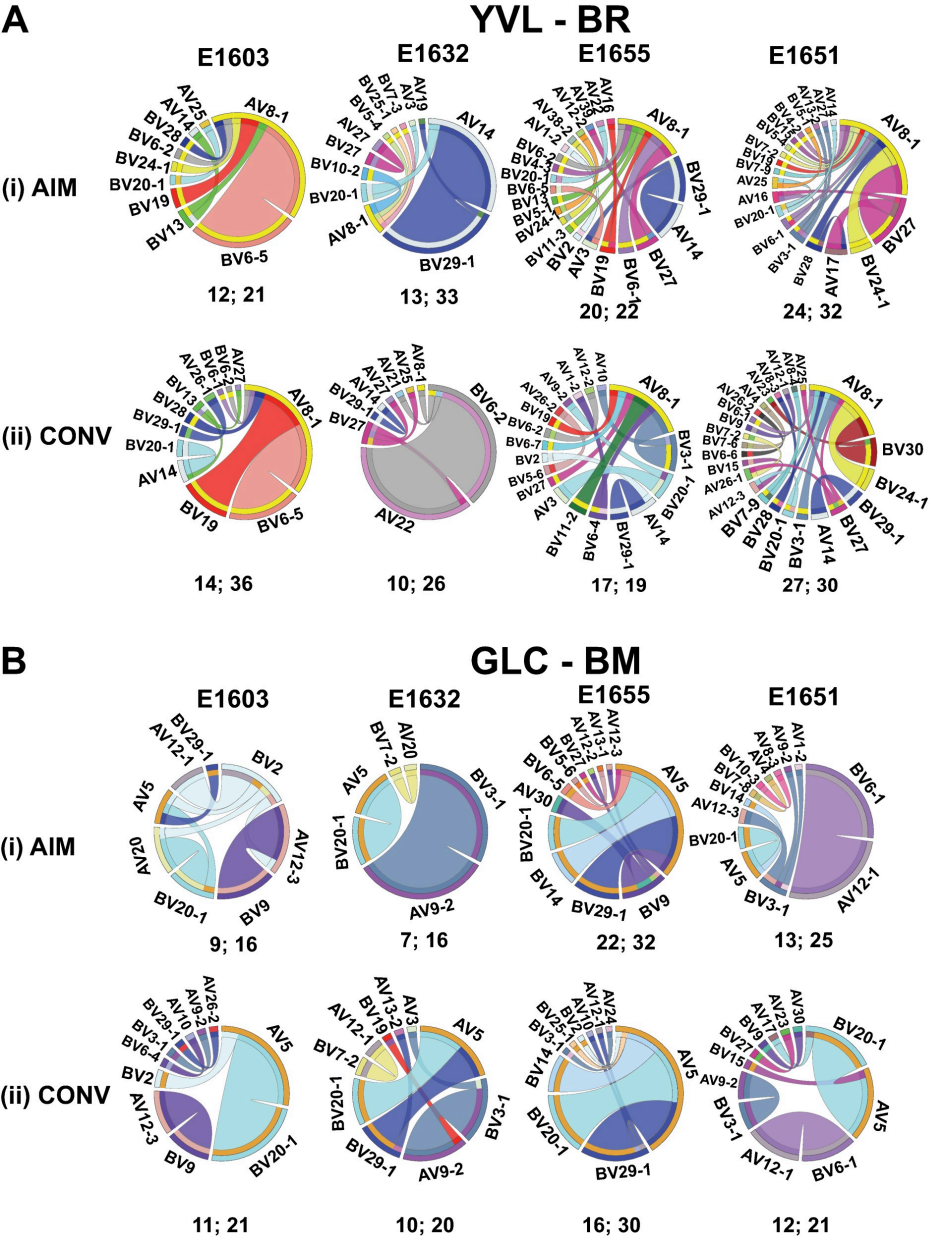
Patterns of AV-BV pairings by YVL-BR (A) and GLC-BM (B) specific CD8 T-cells as revealed by single-cell TCRαβ sequencing. The frequencies of AV-BV combinations in four donors during AIM (i) and CONV (ii) for YVL-BR- (A) and GLC-BM-specific (B) TCRαβ repertoires are displayed in circos plots, with frequency of each AV or BV cassette represented by its arc length and that of the AV-BV cassette combination by the width of the joining ribbon. The numbers of unique and productive paired TCRαβ clonotypes as well as the total numbers of sequences for each donor are shown below the pie charts (# of unique TCRαβ clonotypes; total # of sequences).

To better understand the EBV-specific CD8 TCR repertoire, we performed single-cell paired TCR sequencing of tetramer-sorted, epitope-specific CD8 T cells from four donors during AIM and CONV. A total of 65 and 64 (YVL-BR; AIM and CONV) and 48 and 52 (GLC-BM; AIM and CONV) productive paired TCRαβ sequences were generated (**Tables 1 and S2**). Circos plots were used to examine pairing relationships between AV and BV gene segments by individual, epitope and time point (**Fig 1**). These analyses revealed that each individual had a unique repertoire. For example, the pattern of AV-BV pairing for YVL-BR TCRs was more intricate in some individuals (E1655 and E1651), as demonstrated by the dense interaction web, than in others (E1603 and E1632) (**Fig 1A**). However, despite the uniqueness of the repertoire, there were prominent features shared across individuals and these features were peculiar to each epitope. Such features included the use of AV8.1 by YVL-BR TCRs in all individuals in both AIM and CONV. This gene was overrepresented in three of the four donors (E1603, E1655, and E1651) and paired with multiple BV genes **(Fig 1A)**. The pairing of AV8.1 with multiple BV genes was more pronounced in E1651 and E1655. The BV repertoire of YVL-BR used multiple different families that differed between the donors with no obvious shared features. With respect to GLC-BM, although there were difference between the donors, AV5 and BV20 were common in all four patients in AIM and CONV. The public AV5-BV20 pairing was conserved in all individuals **(Fig 1B),** consistent with previous reports [31, 32].

**Table 1:** Paired single-cell YVL-specific TCR amino acid sequences.

	Color-coded by CDR3α clones	Color-coded by CDR3β clones	Count	Count
Donor ID	CDR3α	CDR3β	AIM	CONV
E1632	AV8CAVKDTDKLIF AJ34	BV10CASMLPFGDEQYF BJ1	1	
E1632	AV8CAVKDTDKLIF AJ34	BV10CASMLPFGDEQYF BJ2	1	
E1655	AV8CAAPGAGSYQLTF AJ28	BV11CASMRELAGQETQYF BJ2		2
E1655	AV8CNASGAGSFHFTF AJ28	BV11CASMRELAGQETQYF BJ2	1	
E1603	AV14CAMREGTGNFNKFYF AJ21	BV13CASRQTSGELFF BJ2	1	1
E1603	AV27SSPRFSDGQKLLF AJ16	BV13CASRQTSGELFF BJ2		1
E1603	AV8CAVKGGGADGITF AJ45	BV13CASRQTSGELFF BJ2	1	
E1655	AV8CLIGQAGFTLIF AJ15	BV13CASSSPRGTGGRDTGELFF BJ2	1	
E1651	AV12CAMSASNFGNEKLTF AJ48	BV15CATSSTARDSSYSNQPQHF BJ1		1
E1651	AV8CALSGGSQGNLIF AJ42	BV15CATSTGLAGIHEQYF BJ2	1	
E1603	AV8CAVKDTDKLIF AJ34	BV19CASIAYLGSNQPQHF BJ1	1	
E1655	AV8CAVNVPDGQKLLF AJ16	BV19CASRALLGGATEAFF BJ1	1	1
E1603	AV14CAMRGGVNNHNKFIF AJ21	BV19CASRTAGNSDTQYF BJ2	1	
E1603	AV8CAVKDTDKLIF AJ34	BV19CASRTAGNSDTQYF BJ2	1	10
E1655	AV16CGVRNRDDKIIF AJ30	BV19CASSIGFDIETQYF BJ2	1	
E1651	AV8CAVKDTDKLIF AJ34	BV19CASSSLLISEAFF BJ1	1	
E1603	AV14CAMREGGNFNKFYF AJ21	BV20CSAAQALYNEQFF BJ2		1
E1655	AV3CAVREGGNFNKFYF AJ21	BV20CSAGQALRNEQFF BJ2		1
E1603	AV14CAMREGGNFNKFYF AJ21	BV20CSAGQVLEQPQHF BJ1		1
E1651	AV12CAMSASNFGNEKLTF AJ48	BV20CSANDRSYNEQFF BJ2		1
E1655	AV3SVVSTGARFNKFFF AJ21	BV20CSARDLAGNTGELFF BJ2		1
E1655	AV9CALRDTSGSRLTF AJ58	BV20CSARDSRDLLRGYTEAFF BJ1		1
E1651	AV25CAAGSNDYKLSF AJ20	BV20CSARGTLFYEQYF BJ2	1	
E1651	AV8CAVNDNARLMF AJ31	BV20CSARGTSFYEQYF BJ2		1
E1603	AV25CAGSSNDYKLSF AJ20	BV20CSARGTTFYEQYF BJ2	1	
E1655	AV22CAVGPLVRF AJ43	BV20CSASSNSAYGYTF BJ1	1	
E1632	AV14CAMREGGNFNKFYF AJ21	BV20CSASYPAGLQAGGGDEQYF BJ2	2	
E1651	AV14CAMRAGGNFNKFYF AJ21	BV20CSATIPPDNEQFF BJ2	1	
E1632	AV3CAVRDGGNFNKFYF AJ21	BV20CSAVGLAGGFIVDEQFF BJ1	1	
E1632	AV3CAVRDGGNFNKFYF AJ21	BV20CSAVGLAGGFIVDEQFF BJ2	1	
E1603	AV14CAMREGGNFNKFYF AJ21	BV20CSGGQALHNEQFF BJ2		1
E1603	AV8CAVKDTDKLIF AJ34	BV24CATSDAHVNEQFF BJ2	1	
E1651	AV8CAVNAHTDKLIF AJ34	BV24CATSDFLSNEQFF BJ2		1
E1655	AV38CASLNSGGGADGLTF AJ45	BV24CATSDPTRGRRNNEQFF BJ2	1	
E1651	AV8CAVKDTDKLIF AJ34	BV24CATSDWDDSTGELFF BJ2	4	2
E1651	AV8CAVKNQAGTALIF AJ15	BV24CATSDWDDSTGELFF BJ2		1
E1632	AV8CAVKNQAGTALIF AJ15	BV25CASSEWGLGEAFF BJ1	1	
E1632	AV25CAGATIGFGNVLHC AJ35	BV27CASGRLAGYNEQFF BJ2		1
E1632	AV22WAAGSGVGSFQLTF AJ28	BV27CASSFLGQTMNTEAFF BJ1		1
E1632	AV27CAGPLTGKLIF AJ37	BV27CASSFLGQTMNTEAFF BJ1	3	1
E1655	AV39CAVMRTYKYIF AJ40	BV27CASSLRGGGYEQYF BJ2	1	
E1651	AV26CIVRGRNFGNEKLTF AJ48	BV27CASSLSITRVYEQYF BJ2		1
E1651	AV8CAVKDTDKLIF AJ34	BV27CASSLSMTHTYNEQFF BJ2	1	
E1655	AV8CAVKQTDKLIF AJ34	BV27CASSPDIGWGGEQFF BJ2	1	
E1655	AV8CAVKDTDKLIF AJ34	BV27CASSPSLSDYFTDTQYF BJ2		1
E1651	AV16CALRGSGNQFYF AJ49	BV27CASSPTTMLETQYF BJ2	1	
E1651	AV8CAVKDTDKLIF AJ34	BV27CASSRAASSSYNEQFF BJ2		1
E1651	AV25CAGLQGANNIFF AJ36	BV27CASSRDASSSYNEQFF BJ2		1
E1651	AV8CAVKDTDKLIF AJ34	BV27CASSRDASSSYNEQFF BJ2	3	
E1651	AV8CAVKGTYKYIF AJ40	BV27CASSSNPMLETQYF BJ2	2	
E1651	AV8SIGKGPDKLIL AJ34	BV27CASSSNPMLETQYF BJ2	1	
E1651	AV17CATDADYGQNFVF AJ26	BV27CASSTVPGHQPQHF BJ1	3	
E1655	AV8CALPGLNNDMRF AJ43	BV27CASSTYSGRATEQYF BJ2	1	
E1603	AV8CAVNNQAGTALIF AJ15	BV28CAGRPLLGGGSPLHF BJ1		1
E1651	AV8CAVIAGGYQKVTF AJ13	BV28CANVMGGPEGGYTF BJ1	1	
E1603	AV8CALKDTDKLIF AJ34	BV28CASRPWGGTGELFF BJ2		1
E1603	AV8CAVKNQAGTALIF AJ15	BV28CASRSSYNSPLHF BJ1	1	
E1651	AV17CALNTGGFKTIF AJ9	BV28CASSLSGSRSEQFF BJ2	1	
E1651	AV8CAVKDTDKLIF AJ34	BV28CASSSYPGLSTGELFF BJ2	1	
E1651	AV8CAVSNTDKLIF AJ34	BV28CASSSYPGLSTGELFF BJ2		1
E1651	AV8CAVAGTGNQFYF AJ49	BV28CPAVPKGDSAPGTVFF BJ2		1
E1632	AV14CAMREGGNFNKFYF AJ21	BV29CSVAKPAGLAGGSNTGELFF BJ2	6	
E1632	AV14CAMREGGNFNKFYF AJ21	BV29CSVGGTGSGTSGAYSYNEQFF BJ2	13	1
E1632	AV14GAMREGGNFNEFYC AJ21	BV29CSVGGTGSGTSGAYSYNEQFF BJ2	1	
E1651	AV14CAMREGGNFNKFYF AJ21	BV29CSVGGTSGSVSYNEQFF BJ2		1
E1655	AV14CAMREGGNFNKFYF AJ21	BV29CSVGQALYNEQFF BJ2	1	
E1651	AV14CAMREGGNFNKFYF AJ21	BV29CSVLDPTFSYNEQFF BJ2		1
E1632	AV19CALSSNARLMF AJ31	BV29CSVREAANYGYTF BJ1	1	
E1655	AV14CAMREGGNFQKLVF AJ8	BV29CSVVAGANNEQFF BJ2	2	
E1655	AV14CAMREGGNFNKFYF AJ21	BV29CSVVAPLWNEQFF BJ2	1	
E1603	AV26CIVRVGGNFNKFYF AJ21	BV29CSVVEPPYNEQFF BJ2		1
E1651	AV14CAMREGGNFNKFYF AJ21	BV29CSVVTAPLTEQFF BJ2		1
E1655	AV14CAMREGGNFNKFYF AJ21	BV29CSVVWALGQPQHF BJ1	1	1
E1655	AV14KSYGGGASYGKLTF AJ52	BV29CSVVWALGQPQHF BJ1		1
E1655	AV3CAVNNARLMF AJ31	BV29CSVVWALGQPQHF BJ1	1	
E1603	AV26CIVRVGGNFNKFYF AJ21	BV29PSVVEPPYNEQFF BJ2		1
E1655	AV1CAVRRGSTLGRLYF AJ18	BV2CASSAPGGTGGRNTEAFF BJ1	1	
E1655	AV14CAMSAPPTSGSVRQLTF AJ22	BV2CASSSPLAESPAGELFF BJ2		1
E1651	AV8CAVKDTDKLIF AJ34	BV30CAWSGRPSYNEQFF BJ2		2
E1651	AV8CAVKATDKLIF AJ34	BV30CAWSGTPIYNEQFF BJ2		2
E1651	AV23CAASIPHFQAGTDLIF AJ15	BV30CAWSGTSPSSSYNEQFF BJ2		1
E1651	AV8CAVAGTGNQFYF AJ49	BV30CSGRGQSTSEQYF BJ2		1
E1651	AV8CAVKNTDKLIF AJ34	BV3CASSQDLGQIETQYF BJ2		1
E1655	AV10CVVSAPTGANKLIF AJ20	BV3CASSQDRAAYGYTF BJ1		1
E1651	AV8CAQGDAGNMLTF AJ39	BV3CASSQEPGSGETQYF BJ2		1
E1603	AV8CAVNNAGNMLTF AJ39	BV3CASSQEPLSGDTQYF BJ2	1	
E1655	AV8CAVGNTGKLIF AJ37	BV3CASSQGISSGNTIYF BJ1		2
E1651	AV13CAERRGGNFNKFYF AJ21	BV3CASSQGLADGETQYF BJ2	1	
E1651	AV27CARGNEKLTF AJ48	BV3CASSQGLSGRAHEQFF BJ2	1	
E1651	AV8CAVSSAGKSTF AJ27	BV3CASSQGPSSGNTIYF BJ1	1	
E1655	AV8CAVKDTDKLIF AJ34	BV3CASSQVIGVGYTF BJ1		1
E1655	AV8CAFGGYNKLIF AJ4	BV4CASSDVTGTYGYTF BJ1	1	
E1651	AV8CAVKDTDKLIF AJ34	BV4CASSNTAGVLGDEQFF BJ2	1	
E1655	AV26CILRDSHTGTASKLTF AJ44	BV5CASKSLSSYEQYF BJ2		1
E1655	AV12CAVGNTNAGKSTF AJ27	BV5CASSLAAREDEQYF BJ2	1	
E1651	AV25CAYYNFNKFYF AJ21	BV5CASSLELAGANSYEQYF BJ2	1	
E1632	AV8CAGYNFNKFYF AJ21	BV5CASSLFSGNEQFF BJ2	1	
E1651	AV8CAVIFFNKFYF AJ21	BV5CASSLGASGSSEQFF BJ2	1	
E1655	AV8CAVKDTDKLIF AJ34	BV6CARSGTSDTLSYNEQFF BJ2		1
E1603	AV8CALNSNYQLIW AJ33	BV6CASRALSGGGQPQHF BJ1		1
E1632	AV22CAAGSGAGSYQLTF AJ28	BV6CASRHPLGGASEQYF BJ2		1
E1651	AV8CAVNSDYKLSF AJ20	BV6CASRQLTGGAQPQHF BJ1		1
E1632	AV8CAVGNYQLIW AJ33	BV6CASRQQGSTEAFF BJ1		1
E1603	AV8CAGKGGGADGLTF AJ45	BV6CASSALLGSASTQYF BJ2	1	
E1603	AV8CAVKGGGADGLTF AJ45	BV6CASSALLGSASTQYF BJ2	10	14
E1655	AV1CAVNNDYKLSF AJ20	BV6CASSASPGGAGNEQFF BJ2		1
E1655	AV8CAVKDTDKLIF AJ34	BV6CASSDTLSTGELFF BJ2		1
E1651	AV16CALKDTDKLIF AJ34	BV6CASSEFSMYEAFF BJ1	1	
E1603	AV8CAFYHAGNMLTF AJ39	BV6CASSGPGWDEQYF BJ2	1	
E1655	AV3CAVNNARLMF AJ31	BV6CASSPDSYEQYF BJ2	1	
E1655	AV8CAVKDTDKLIF AJ34	BV6CASSSFRDSSNEQYF BJ2	2	
E1655	AV12CAVNGPPPSGSARQLTF AJ22	BV6CASSSTYPGSVGETQYF BJ2	1	
E1603	AV8CAVKDTDKLIF AJ34	BV6CASSSVLDFLGTGELFF BJ2	1	
E1655	AV8CAVKDTGKLIF AJ37	BV6CASSVIDTQYF BJ2	1	
E1651	AV8CAVKDTDKLIF AJ34	BV6CASSVPTLSDGYTF BJ1	1	
E1632	AV21CAVPMYSGGGADGLTF AJ45	BV6CASSYIGVGYTF BJ1		1
E1632	AV22CAAGSGAGSYQLTF AJ28	BV6CASSYPTGHSSYNEQFF BJ2		17
E1632	AV22CAAGSGVGSYQLTF AJ28	BV6CASSYPTGHSSYNEQFF BJ2		1
E1632	AV22RDAGCGAGSYQFTF AJ28	BV6CASSYPTGHSSYNEQFF BJ2		1
E1603	AV8CAVKDTDKLIF AJ34	BV6CASSYVGLLGDTQYF BJ2		1
E1651	AV26CILRGPPLGNEKLTF AJ48	BV6CASTGIQGNTGELFF BJ2		1
E1651	AV8CAVNAVGDMRF AJ43	BV7CASSAGQGSGNTIYF BJ1	1	
E1651	AV26CIVPSTSGTYKYIF AJ40	BV7CASSLAGINYGYTF BJ1		1
E1632	AV8CAVKNQAGTALIF AJ15	BV7CASSPDPTGYNEQFF BJ2	1	
E1651	AV8CAVKNQAGTALIF AJ15	BV7CASSQGPTGDTQYF BJ2	1	
E1651	AV8CAVSEGTNAGKSTF AJ27	BV7CASSYTGRALEAFF BJ1		1
E1651	AV8CAVVTGGFKTIF AJ9	BV7CASSYTTGSADTQYF BJ2		1
E1651	AV12CVVNRLDNAGNMLTF AJ39	BV9CASSVAGTSVETQYF BJ2		1

### The selection of the YVL-BR-specific repertoire is mainly driven by TCRα

**Fig 2:**
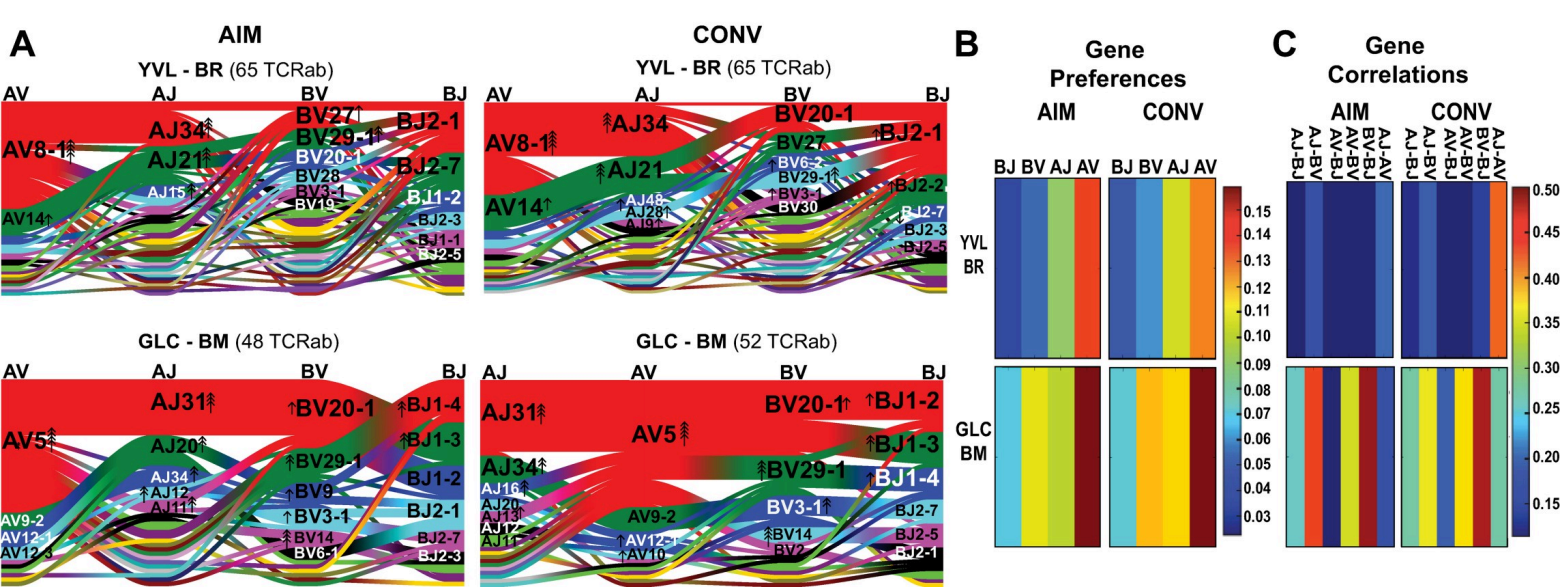
Single-cell paired TCRαβ sequencing provides evidence that the selection of YVL-BR-specific repertoire is mainly driven by TCRα. **(A)** Gene segment usage and gene–gene pairing landscapes for all four donors, combined, during AIM and CONV for YVL-BR- (Top) and GLC-BM-specific (Bottom) TCRαβ repertoires are illustrated using four vertical stacks (one for each V and J segment) connected by curved paths whose thickness is proportional to the number of TCR clones with the respective gene pairing (each panel is labeled with the four gene segments atop their respective color stacks and the epitope identifier in the top middle). Genes are colored by frequency within the repertoire, which begins red (most frequent), green (second most frequent), blue, cyan, magenta, and black. The enrichment of gene segments relative to background frequencies is indicated by up or down arrows with an arrowhead number equal to the log2 of the fold change. (B), Jensen–Shannon divergence between the observed gene frequency distributions and background frequencies, normalized by the mean Shannon entropy of the two distributions (higher values reflect stronger gene preferences). (C), Adjusted mutual information of gene usage correlations between regions (higher values indicate more strongly covarying gene usage). Analyses are based on Dash *et*. *al*. [20].

The qualitative analysis of the repertoire discussed above suggested the presence of generalizable features associated with epitope-specific TCRs. To quantitatively evaluate these features, we used a TCRαβ sequence-based analytical tool recently developed by Dash and colleagues [20]. This tool identifies features that are significantly enriched in an epitope-specific repertoire by quantifying different properties of an epitope-specific TCR repertoire and comparing them against a collection of publicly available human TCR sequences. The Shannon-Jensen divergence is used to identify significantly enriched gene segments (**Fig 2A**) and to score the magnitude of preferential gene usage (**Fig 2B**) within an epitope-specific repertoire, then the adjusted mutual information is used to measure the magnitude of gene usage correlations within and across TCRα/β (**Fig 2C**). In order to work with a large dataset, we merged the data from all patients, per time point and per epitope. We first examined the pattern of VA-JA/VB-JB gene segment pairing by ribbon plots (**Fig 2A**). There were identifiable enriched gene segments and pairings. For example, the AV8.1/AJ34 pairing was significantly enriched in the YVL-BR repertoire in AIM and CONV but paired with multiple different BV/JB genes (**Fig 2A**). In contrast, the AV5/AJ31 combination paired with BV20.1/ BJ1.3 was significantly enriched in the GLC-BM repertoire in AIM and CONV (**Fig 2A**).

To quantitatively evaluate the degree of bias in the repertoire, we evaluated the gene preference score. We observed that YVL-BR-specific CD8 T cells exhibited a strong preferential usage of particular AV and AJ in both AIM and CONV. By contrast, GLC-BM-specific TCRs exhibited strong preferential usage of particular AV, AJ, BV and BJ **(Fig 2B)**. This is consistent with previous structural studies demonstrating that both the TCR α and β chains are important for recognition of the GLC-BM epitope [33]. Altogether, these data suggest that the TCRα chain may be important for selection of YVL-BR-specific CD8 T cells whereas both the TCR α and β chains together are important for selection of GLC-BM-specific CD8 T cells. Moreover, quantification of the degree of gene usage correlations within and across TCRα/β revealed that GLC-BM-specific TCRα and β repertoires were rigid; every gene association except for two in AIM (AV-AJ and AV-BJ pairings) and one in CONV (AV-BJ) were enriched and thus important for selection into the repertoire **(Fig 2C)**. In contrast, the YVL-BR repertoire was highly flexible in gene pairings. No obvious gene associations emerged for YVL-BR-specific TCR in AIM, whereas only the AV-AJ association emerged as important in CONV **(Fig 2C)**. These data highlight the differences in selection of the YVL-BR and GLC-BM TCR repertoires at the clonal level.

### Identification of a shared public and dominant CDR3α, AV8.1-KDTDKL-AJ34 that pairs with multiple different BV genes within the YVL-BR repertoire

**Fig 3:**
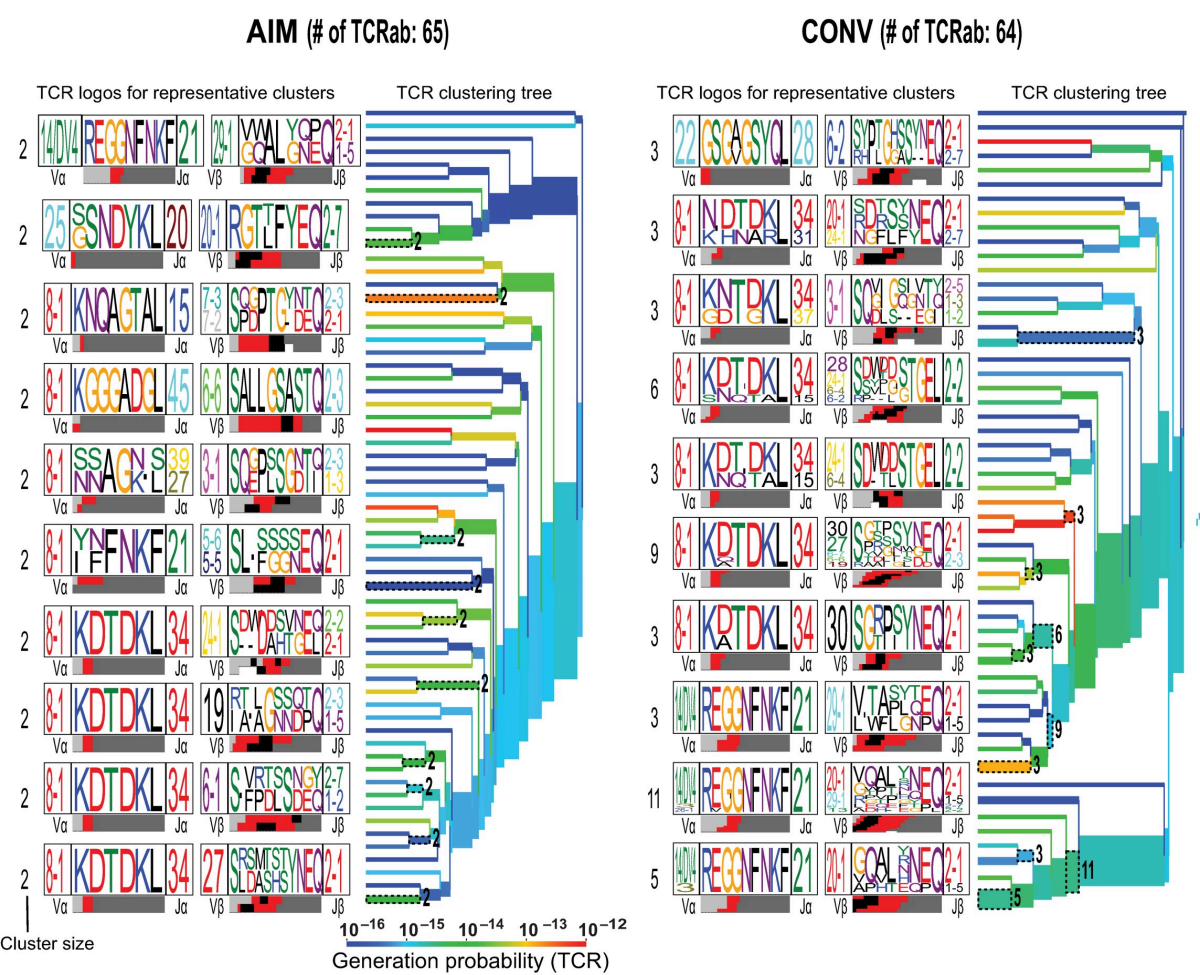
Hierarchical clustering of TCRs highlights the structural features required for interaction with pMHC of paired TCRα/β. (**A-B)** Hierarchical TCRαβ clustering along with corresponding TCR logos for YVL-BR-specific CD8 T-cell responses in AIM **(A)** and CONV **(B).** Number on the branches and next to TCR logos depicts number of TCRs contributing to the cluster. Color of the branches indicates the TCR probability generation scores. The bar at the bottom of the CDR3 logo is color-coded by the source of the nucleotide. Light grey, red, black, and dark grey denote that the nucleotides encoding those amino acid residues originate from the V, N, D and J regions, respectively. Analyses are based on Dash *et al*. [20].

**Fig 4:**
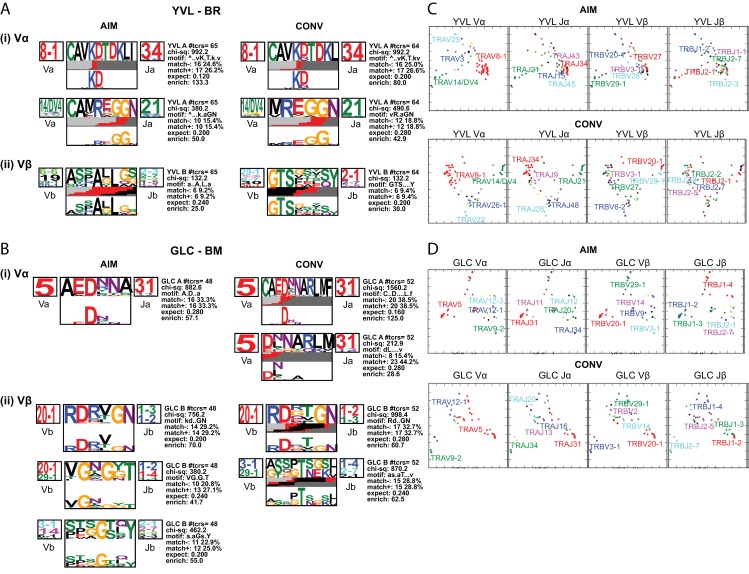
Single-cell CDR3α (i) and β (ii) motif analyses of YVL-BR- (A) and GLC-BM- (B) specific CD8 T cells during AIM and CONV. CDR3α or β sequences were pooled from all 4 patients during AIM and CONV. Using TCRlogo in TCRdist algorithm, top-scoring CDR3β and CDR3α motifs are shown for each group. V and J gene usage are indicated on the left and right side of TCR logos, respectively. The top logo is based on comparing clusters of similar clonotypes comparing where the CDR3 tolerates or does not tolerate mutations or alternate amino acids. The bottom panel highlights the enriched amino acids within the motif by calculating against a background dataset of naïve non-YVL-BR or GLC-BM-specific CD8 TCRs. The bar at the bottom of the CDR3 logo is color-coded by the source of the nucleotide. Light grey, red, black, and dark grey denote that the nucleotides encoding those amino acid residues originate from the V, N, D and J regions, respectively. **(C-D)** 2D kernel principal component analysis (kPCA) projections for YVL-BR (C) and GLC-BM (D) specific responses (*n* = 4 donors pooled in AIM and CONV). Color correlates with gene usage. Most prevalent gene usages are mentioned within the plots matching with clonotype color. Each row represents group and each column is the same 2D kPCA projection of the four gene segment usage (Vα, Jα, Vβ, and Jβ). Analyses are based on Dash *et al*. [20].

To dissect the clonal composition of the TCRαβ repertoires, we clustered similar clones into groups **(Figs 3 and 4)** by performing a number of analyses including 2D kernel principal component analysis (kPCA) projections (**Fig 4C–4D**) and hierarchical clustering with dendograms (**Figs 3 and S3**) for YVL-BR (C) and GLC-BM (D) specific responses (*n* = 4 donors pooled in AIM and CONV). In the 2D kPCA projections, the color correlates to gene usage. The hierarchical clustering is presented as a dendogram of the paired TCRαβ clones and also derived TCR logo representations showing gene usages and frequencies and CDR3 amino acid sequences of specific clusters **(Figs 3 and 4C and S3)**. For the YVL-BR response, clustering was driven by the TCRα chain, particularly the dominant AV8.1-KDTDKL-AJ34 expressing clones; this TCRα chain was detected in all individuals and resulted from an obligate pairing between AV8.1 and AJ34 (**Fig 3**). More importantly, this public AV8.1-KDTDKL-AJ34 TCR is so important for selection of the YVL-BR TCR repertoire that there is an unusually high frequency of clones where this one TCRα chain pairs with multiple different TCRβ chains within a single donor (median 4; range: 1–9) (**Fig 3 and Table 2**). It is not uncommon to find a single TCRβ chain to rearrange and pair with multiple different TCRα as TCRβ rearranges first and is expressed before TCRα. Because of this order in TCR rearrangement, it would be less common to see multiple TCRβ with the same TCRα. This finding suggests that this TCRα is so highly favored by its interaction with EBV-BR/MHC that these rare event TCR rearrangements dominate the repertoire. In contrast, in the GLC-BM TCR repertoire there was no evidence of such pairing of a single public TCRα chain being paired with multiple different TCRβ chains or vice versa. Unlike YVL-BR, the clustering of GLC-BM-specific TCRs was driven by dominant interactions with both the TCRα and β chains **(Figs 4D and S3)**.

**Table 2:** TCR AV8-CAVKDTDKLIF-AJ34 pairs with multiple different TCRβ within the same individual.

Donor ID	AV	CDR3α sequence (AA)	AJ	CDR3α length	BV	CDR3β sequence (AA)	BJ	CDR3β length
E1603	AV08	AVKDTDKLI	AJ34	9	BV24	ATSDAHVNEQF	BJ02	11
					BV06	ASSYVGLLGDTQY	BJ02	13
					BV06	ASSSVLDFLGTGELF	BJ02	15
					BV19	ASRTAGNSDTQY	BJ02	12
E1632	AV08	AVKDTDKLI	AJ34	9	BV10	ASMLPFGDEQY	BJ01	11
E1651	AV08	AVKDTDKLI	AJ34	9	BV30	AWSGRPSYNEQF	BJ02	12
					BV24	ATSDWDDSTGELF	BJ02	13
					BV06	ASSVPTLSDGYT	BJ01	12
					BV28	ASSSYPGLSTGELF	BJ02	14
					BV19	ASSSLLISEAF	BJ01	11
					BV27	ASSRDASSSYNEQF	BJ02	14
					BV27	ASSRAASSSYNEQF	BJ02	14
					BV04	ASSNTAGVLGDEQF	BJ02	14
					BV27	ASSLSMTHTYNEQF	BJ02	14
E1655	AV08	AVKDTDKLI	AJ34	9	BV06	ASSSFRDSSNEQY	BJ02	13
					BV03	ASSQVIGVGYT	BJ01	11
					BV27	ASSPSLSDYFTDTQY	BJ02	15
					BV06	ASSDTLSTGELF	BJ02	12
					BV06	ARSGTSDTLSYNEQF	BJ02	15

### Predictable CDR3α motif features drive the selection of the shared AV8.1-KDTDKL-AJ34 expressing clones

To better understand the factors driving the selection of AV8.1-KDTDKL-AJ34 expressing clones, we analyzed CDR3 sequences for motifs and conserved residues that may determine epitope recognition [20]. The analytical tool reported by Dash and colleagues [20] is a predictive algorithm for the identification of key enriched residues within CDR3 regions that may be important in antigen recognition, known as the CDR3 motif. It is based on the principle that amino acids within the CDR3 which do not tolerate substitutions when comparing clusters of similar TCRα or β chain sequences must be important for interaction with the pMHC. It also identifies sequence patterns or motifs that occur more frequently in an epitope-specific TCR repertoire compared with a background dataset, which consists of publicly available and randomly selected unpaired non-epitope-specific TCRs derived from high-throughput TCR repertoire profiling [34–37] and calculates fold enrichment of the observed motifs in epitope-specific repertoires over the expected in the background dataset **(Fig 4A and 4B)**.

With this useful algorithm, for GLC-BM we identified a known [20, 33] highly public dominant CDR3α motif AEDNNA, with a conserved Asp(D) residue within these public AV5-AJ31 expressing clones (**Fig 3Bi**). We also identified two known [20, 33] CDR3β motifs, RDxTGN within the public BV20.1-expressing clones in CONV and S/P,T/P,S/G,G within the public BV14-expressing clones in AIM (**Fig 3Bii**) as being important for GLC-BM recognition. We should note that CDR3 motifs that were identified within the GLC-BM response during AIM used similar clonotypes in CONV, particularly the highly public AV5-AEDNNA-J31 paired with VB20.1-SARDXXGN-J1.2/1.3, consistent with observations from deep sequencing data of a strong selection of these particular clonotypes into CONV (Gil *et al*., manuscript submitted, 2019).

In the YVL-BR-specific TCR repertoire, we discerned a strong CDR3α motif and some CDR3β motifs **(Fig 4A and 4C)**. Within the public AV8.1/AJ34 clonotypes, the CAVKDTKL motif was highly conserved **(Fig 4A)** from AIM to CONV and contained the conserved amino acid pair “KD” which is partially non-germline **(S2 Fig)**, suggesting that they may provide a selective advantage and play a critical role in YVL-BR recognition. There was a second CDR3α motif (CAXRxGGN) that included a conserved “GG” pair predominantly resulting from the AV14-AJ21 recombination event. Interestingly, although many different BV were used in the YVL-BR response, this algorithm identified two potential CDR3β motifs, “xAxL”, present in AIM and “GTSx” present in CONV, resulting from multiple BV-BJ recombination events; this suggests that TCRβ may play some role in selection of YVL-BR-specific TCRs but it differs between AIM and convalescence.

**Fig 5:**
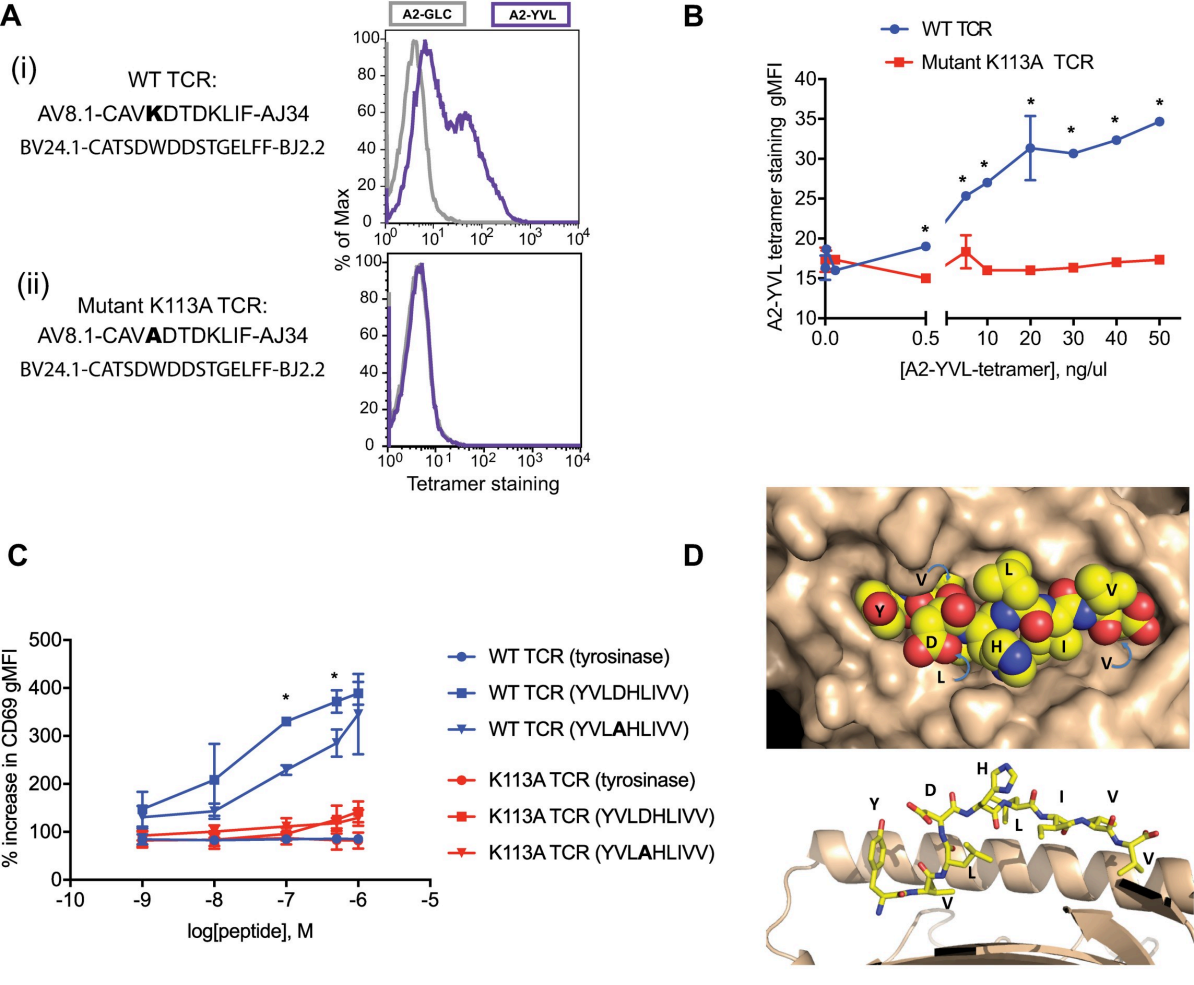
Proposed mechanism for the selection of the public AV8.1-CAVKDTDKLIF-AJ34 clonotype. (A) Tetramer staining of J76-cells transduced with either the WT (i) or mutant K113A TCR (ii) and stained with the cognate A2-YVL-BR (purple) or an irrelevant A2-GLC-BM (grey) tetramer. CDR3α/β sequences are noted below each TCR name. The bold residue in the CDR3α denotes the one that has been mutated. (B) Tetramer titration of J76-cells transduced with either the WT (blue) or mutant K113A TCR (red). Assay was performed in three technical replicates and mean ± SD is shown. (C) CD69 upregulation of J76-cells transduced with either the WT (blue) or mutant K113A TCR (red). The cells were stimulated with T2-cells pulsed with the respective peptides at different concentrations (10^−9^, 10^−8^, 10^−7^, 5 x 10^−7^ and 10^−6^ M). HLA-A2-restricted epitope derived from tyrosinase protein (YMDGTMSQV) was used as a negative control. Amino acid sequences of the WT and mutant BRLF-1_109−117_ peptides are shown in parentheses. The bold residue in the peptide sequence denotes mutated residue (D4A mutant peptide). Assays were performed in two technical and biological replicates and mean ± SD is shown. (D) Crystal structure of YVL-BR pMHC. *Top*, top view (or TCR-view) of HLA-A2/YVL-BR complex, with peptides atoms shown as spheres and HLA-A2 surface in tan. Peptide sequence indicated in single letter code. *Bottom*, cut-away side view of HLA-A2/YVL-BR complex with peptide in stick model and HLA-A2 as a ribbon. The alpha-2 helix in front of the peptide was removed for clarity. Statistical analyses were done using multiple t tests. *: p value < 0.01. WT: wild-type.

To experimentally test whether the first Lys(K) in the CAVKDTKL motif and present in the highly conserved amino acid pair, “KD” was important for YVL-BR recognition, we cloned one of the YVL-BR-specific TCR expressing the AV8.1-CAVKDTKL-AJ34 motif (wild-type, WT,TCR; AV8.1-CAVKDTDKLIF-AJ34BV24.1-CATSDWDDSTGELFF-BJ2.2). We confirmed the antigenic specificity of the cloned WT TCR by expressing it in TCR-null CD8α-expressing J76 cells; the TCR-transduced cells stained with YVL-BR tetramer (**Fig 5Ai and 5B)**. To determine whether the Lys(K) within the conserved “KD” amino acid pair was involved in antigen recognition, we mutated the WT TCR (K113A TCR). This mutation abrogated YVL-BR tetramer staining (**Fig 5Aii and 5B**), consistent with this Lys(K) contributing to YVL-BR/MHC recognition. We confirmed that the mutated TCR was expressed by measuring CD3 upregulation (**S1 Fig**), which excludes the possibility that the lack of tetramer staining was due to a lack of TCR expression. Increasing the amount of YVL-BR tetramer did not result in tetramer staining to the mutated TCR (K113A; **Fig 5B**). Additionally, we observed functional signaling through the wild-type TCR as measured by the upregulation of CD69 following stimulation of the WT TCR-transduced J76 cells with peptide-pulsed HLA-A2-expressing T2 cells acting as antigen presenting cells **(Fig 5C)**. Introduction of the K113A mutation substantially decreased CD69 upregulation, further indicating that Lys(K) contributed to YVL-BR/MHC recognition **(Fig 5C)**. Of interest, a small functional response was still inducible even though tetramer no longer bound to this mutated TCR, consistent with previous studies demonstrating induction of functional response but no tetramer binding when using variant ligands [17, 38–40]. To further dissect the role of the Lys(K) within the KD motif, we investigated the effect of D4→A peptide mutation (D4A mutant peptide) on CD69 upregulation on cells expressing the WT TCR **(Fig 5C)**. We observed that J76 cells expressing the WT TCR and stimulated with T2 cells pulsed with either the WT peptide or the D4A mutant peptide displayed levels of CD69 upregulation not significantly different from background levels (cells stimulated with a non-cognate HLA-A2-restricted epitope derived from the protein tyrosinase) at peptide concentration of 10^−9^ M and 10^−8^ M. However, as peptide concentration increased, CD69 expression on WT TCR-expressing cells stimulated with D4A mutant peptide at peptide concentrations of 5x10^-7^ and 10^−7^ M was significantly reduced compared to those stimulated with the WT peptide. These observations suggest that the Asp(D) residue at position 4 of the peptide plays a role in mediating T-cell signaling. However, the Lys(K) on the CDR3α may play a more important role given that mutation of this residue led to substantial reduction of CD69 upregulation. Further structural analyses of the TCR with its cognate ligand will be necessary to ascertain the existence of an interaction between the Lys(K) in the “KD” CDR3α motif and Asp(D) at position 4 on the peptide.

### Structural features of YVL-BR/MHC support the role of Vα in selection of the YVL-BR-specific TCR repertoire

We next determined the crystal structure of the HLA-A2/YVL-BR complex in order to identify specific structural features that might be associated with the dominant AV8.1-KDTDKL-AJ34 usage in the YVL-BR-specific TCRs (**Fig 5D**). Structure determination was complicated initially by the relatively low resolution (**S3 Table**) and high degree of translational pseudosymmetry (**S4A–S4D Fig**) that characterized the HLA-A2/YVL-BR crystals (see Methods), but ultimately clear and continuous electron density extending the full length of the peptide allowed confident location of all peptide atoms (**S4E and S4F Fig**). In the HLA-A2 complex, the nonameric YVL-BR peptide is bound in conventional orientation, with the amino terminus and side chains of Val(V) at position 2 and Val(V) at position 9 accommodated in pockets (A, B, and F, respectively) in the peptide binding site. Notably, the side chains of Tyr(Y) at position 1, highly negative charged Asp(D) at position 4, and His(H) at position 5 project away from the binding site, and compose a highly featured surface positioned for recognition by CDRα loops of a TCR bound in conventional orientation (**Fig 5D**). By contrast, the side chains of Leu(L) at position 6, Ile(I) at position 7, and Val(V) at position 8, together with exposed main-chain atoms, present a rather non-descript generally hydrophobic surface positioned to interact with CDRβ loops of a TCR bound in conventional orientation.

## Discussion

We report the use of single-cell TCR sequencing on tetramer-sorted EBV-specific CD8 T cells to study the TCR repertoires at the clonal level to a previously unstudied immunodominant, HLA-A2-restricted EBV epitope, YVL-BR, comparing it to GLC-BM repertoires in the same donors. Our studies are unique since they examined both CD8 TCR α and β directly ex vivo from the peripheral blood of individuals during primary EBV infection (AIM) and again 6 months later in convalescence (CONV). Although the TCR repertoire was individualized (*i*.*e*., each donor studied had a unique TCR repertoire), there were prominent public features that were significantly enriched and shared across individuals.

We identified a CD8 T-cell repertoire to a dominant human viral epitope, YVL-BR, that was significantly biased towards TCRα usage based on the CDR3α. We identified a ubiquitous and public TCRα chain, AV8.1-KDTDKL-AJ34, that paired with multiple different TCRβ chains in the same donor. In contrast and consistent with prior reports, GLC-BM-specific CD8 T-cell repertoires were notably skewed in both TCRα and TCRβ usage with a bias towards use of the public AV5-AJ31-BV20-BJ1.2 combination.

Prominent biases have been noted in the TCR repertoire of various common virus-specific CD8 T cells (influenza, cytomegalovirus, hepatitis C virus) [20, 22, 23, 25, 26, 28, 41]. However, most studies to date have only examined the TCRβ chain. The TCRα repertoire has been under-appreciated due to the technical constraints associated with the lack of VA-family specific monoclonal antibodies and the potential for T cells to co-express two α-chains [29, 30]. The apparent selection of a strongly TCRα-driven YVL-BR repertoire suggests that examination of the TCRα repertoire as well as the TCRβ provides a more comprehensive view of an epitope-specific TCR repertoire.

There have been limited reports of the importance of TCRα in viral epitope-specific responses but this appeared to relate to TCR CDR1α interaction with the MHC as observed with the HLA-A*02-restricted yellow fever virus epitope, LLWWNGPMAV which has a biased *TRAV*12.2 usage [42]. HLA-B*35:08 restricted EBV BZLF1-specific responses appear to be biased in both TCRα and TCRβ usage, much like HLA-A2-restricted GLC-BM [43, 44]. There is a strong preservation of a public TCRα clonotype, AV19-CALSGFYNTDKLIF-J34, which can pair with a few different TCRβ chains. TCRα chain motifs have also been described for HLA-A02:01-restricted MI_58-67_ (GIL-M1) IAV-M1, but these appear to make minor contributions to the pMHC-TCR interaction, which is almost completely dominated by CDR3β [26, 30, 45].

The TCR repertoire of the HLA-A02:01-restricted MI_58-67_ (GIL-M1) epitope from influenza A virus is highly biased towards the *TRBV19* gene in many individuals and displays a strong preservation of a dominant xRSx CDR3β motif. Crystal structures of TCR specific to this epitope have revealed that the TCR is β-centric with residues of the TRBV19-encoded CDR1 and CDR2 loops engaging pMHC and the conserved arginine in the CDR3β loop being inserted into a pocket formed between the peptide and the α2-helix of the HLA-A02:01 [26, 46]. The TCRα is not as important as the TCRβ in pMHC engagement and this helps explain the high degree of sequence conservation in the CDR3β and the variability in the CDR3α. Similarly, studies using EBV virus GLC-BM-specific CD8 T cells have documented that TCR-pMHC binding modes also contribute to TCR biases. Miles and colleagues [33] showed that the highly public AS01 TCR, which is specific to the HLA-A*02:01-restricted EBV-derived GLC epitope, was highly selected by the GLC-BM epitope because of a few very strong interactions of its TRAV5- and TRBV20-encoded CDR3 loops with the peptide/MHC.

Given the aforementioned studies, we reasoned that the differences in constraints in the TCR repertoires of YVL-BR and GLC-BM may give a picture of the essential requirements of antigen recognition and that the topology of the pMHC may provide some structural insights into the mechanisms underlying these constraints. This alpha-centricity displayed by the YVL-BR repertoire might be grounded in the fact that the TCRα chain makes more pronounced contact with its ligand, the pMHC, compared to the TCRβ chain. In light of this apparent TCRα bias in the YVL-BR repertoire and the substantial evidence that TCR-pMHC binding modes also contribute to TCR biases, we hypothesized that the bias for TCRα in the YVL-BR repertoire most likely reflects an inherent feature of how the YVL-BR peptide lies in the HLA-A2 groove or the topology of the pMHC. To understand why the YVL-BR epitope drives the selection of this highly conserved public TCRα, we applied the Dash *et al*. algorithm [20] to identify key amino acid residues that might be important for antigen recognition. We uncovered the AV8.1- KDTDKL-AJ34 motif with the highly conserved “KD” amino acid pair within this clonotype as being potentially critical for recognition of the YVL-BR epitope.

These motifs and conserved residues suggest a structural basis for YVL-BR recognition. Our structural analysis revealed that the MHC-bound YVL-BR bulged at position 4, in a region of the peptide that TCRα would have to accommodate, exposing a negatively charged Asp(D). We cloned a TCR expressing the dominant public TCRα chain AV8.1-KDTDKL-AJ34 and using mutagenesis, we provided evidence indicating that the positively charged Lys(K) residue in the CDR3α of the public TCRα chain was important for YVL-BR/MHC recognition. Hence, we propose that the preferential selection of AV8.1-KDTDKL-AJ34, which contains the positively charged (Lys)K in position 1, is potentially driven by an electrostatic interaction between this Lys(K) and the solvent-exposed Asp(D) on the peptide. Future structural analyses of this TCRαβ with its ligand would be important to validate the existence of this electrostatic interaction and to confirm whether the TCRα contributes the majority of contacts with the pMHC. This apparent preference for TCRα may create a large repertoire of different memory TCRβ that could potentially cross-react with other ligands such as IAV-M1_58_, which predominantly interact with TCRBV[17, 20, 26].

In summary, we describe a virus-specific TCR repertoire that is CDR3α-centric and have proposed a structural basis for the selection of a public TCRα chain with a conserved CDR3 motif that seemingly relies on the topology of the pMHC. These studies underscore the importance of studying the TCRα, which has been underappreciated, as well as the TCRβ chains in order to get a complete picture of the determinants of TCR selection. Our findings will contribute to the growing interest and effort focused on the ability to predict the specificity and efficacy of TCR from sequence analyses for the fundamental goal of designing better T-cell therapies [12, 20, 21, 47–49].

## Materials and methods

### Study population

Four individuals (E1603, E1632, E1655 and E1651) presenting with symptoms consistent with acute infectious mononucleosis (AIM) and laboratory studies consistent with primary infection (positive serum heterophile antibody and the detection of EBV viral capsid antigen (VCA)-specific IgM) were studied as described [17]. Blood samples were collected in heparinized tubes at clinical presentation with AIM symptoms (acute phase) and six months later (memory phase). PBMC were extracted by Ficoll-Paque density gradient media.

### Ethics statement

The Institutional Review Board of the University of Massachusetts Medical School approved these studies, and all participants were adults and provided written informed consent.

### Isolation of YVL-BR- and GLC-BM-specific CD8 T-cells by tetramer staining and single-cell sorting directly ex vivo

The percentages of peripheral blood antigen-specific CD8 T-cells were measured by tetramer staining and flow cytometry. Antibodies were purchased from BD Biosciences and included: anti-CD3-FITC, anti-CD4-AF700 and anti-CD8-BV786, 7AAD and PE-conjugated HLA-A*02:01-peptide tetramers (BRLF-1_109−117_: **YVL**DHLIVV, A2-YVL-BR; BMLF-1_280−288_: **GLC**TLVAML, A2-GLC-BM). Tetramers were made in-house and underwent quality assurance, as previously described [50]. Total CD8 T-cells were enriched from PBMCs by positive selection using MACS technology (Miltenyi Biotec, Auburn, CA) according to the manufacturer’s protocol. The cells were then stained with anti-CD3, anti-CD4, anti-CD8, 7AAD, and GLC-BM- or YVL-BR-loaded tetramers for 30 minutes on ice in staining buffer (1% BSA in PBS). Live CD3+, CD8+, and GLC- BM or YVL-BR tetramer+ cells were sorted for single cell sequencing as described below.

### Single-cell cDNA synthesis and paired TCRαβ analysis of EBV-specific CD8 T-cells

To examine TCRα and TCRβ pairing relationships, we conducted an *ex vivo* single-cell analysis of the paired TCRαβ repertoire of YVL-BR and GLC-BM-specific CD8 T-cells from PBMCs of the 4 donors in AIM and CONV. Single tetramer-positive CD8 T cells were sorted into a 384-well plate containing 0.5 ul of lysis buffer, consisting of a mixture of Triton X 100 (Sigma, St. Louis, MO), RNase inhibitor (Clontech, Mountain View, CA), 3’ SMART CDS Primer IIA (Clontech, Mountain View, CA), using the FACSARIA (BD Biosciences, San Jose, CA). Reverse transcription and global amplification of full-length cDNA was performed using the SMARTer kit (Clontech, Mountain View, CA) as described by the manufacturer. The synthesized cDNA library was used as template to amplify CDR3α/β as previously described [51]. Briefly, amplification involves a two-step multiplex PCR using a pool of 20 and 22 forward primers specific to Vα and Vβ gene families and two reverse primers, each specific to Cα or Cβ. The PCR products were purified and individually analyzed by Sanger sequencing. Nucleotide sequences (see S4 Table) were analyzed according to the IMGT/V-QUEST web-based tool [52]. Only productively rearranged CDR3α and CDRβ sequences without stop codons were used for repertoire analyses.

#### Ribbon plots, gene correlations and gene preferences

An analytical tool developed by Dash *et*. *al*. [20] was used to characterize patterns of gene segment usage by ribbon plots, correlate gene usage within a chain (for example, AV-AJ, BV-BJ) and across chains (for example, AV-BV, AV-BJ), and to quantify gene preference usage (the quantification was done by comparing the gene frequencies in our epitope-specific repertoires to those seen in a background set of publicly available non-epitope-selected repertoire using the Shannon diversity index). The analysis was done by combining productive single-cell TCRαβ sequences from the 4 donors at each time point and for each epitope. A total of 65 and 64 (YVL-BR; AIM and CONV) and 48–52 (GLC-BM; AIM and CONV) productive paired TCRαβ sequences were generated.

### EBV DNA quantitation in B cells

B cells were purified from whole blood using the RosetteSep human B-cell enrichment cocktail according to the manufacturer's recommendations (StemCell Technologies, Vancouver BC, Canada). Cellular DNA was extracted using QIAGEN DNeasy Blood & Tissue Kit (Valencia, CA). Each DNA sample was diluted to 5ng/ul and the Roche LightCycler EBV Quantitation Kit (Roche Diagnostics, Indianapolis, IN) was used to quantify EBV DNA copy number in the samples as recommended by the manufacturer. Reactions were run in duplicate. B cell counts in each sample were determined using a previously described PCR assay to quantify the copy number of the gene encoding CCR5 (two copies per diploid cell) [53]. Samples were normalized to B cell counts and EBV DNA copy number was calculated as DNA copy per 10^6^ B cells.

### TCR cloning

Full-length TCRα and TCRβ chains were amplified using the synthesized cDNA (described above) as template, a forward primer (specific to the corresponding Vα or Vβ gene segment identified by single-cell CDR3 sequencing) and a reverse primer specific to the constant region. The alpha chain was linked to the beta chain via the viral P2A sequence for stoichiometric expression and subcloned by homologous recombination into the pscALPS lentiviral vector (a gift from Dr. Jeremy Luban).

### Lentivirus transduction of TCR

293T cells (CRL-3216, ATCC) and J76 cells (a TCR-deficient CD8α-expressing Jurkat cell line [54]; a gift from Dr. Wolfgang Uckert) were maintained in DMEM and RPMI, respectively, containing 10% FBS, HEPES and L-glutamine. Viral packaging was performed in 293T cells using the following three vectors (gifts from Dr. Jeremy Luban): recombinant lentiviral vector containing the cloned TCR gene, an HIV-1 Gag-Pol packaging plasmid (psPAX2) and a VSV-G plasmid (pMD2.G), and the Lipofectamine 2000 Transfection Reagent Kit (Invitrogen, Carlsbad, CA) as recommended by the manufacturer. Infection was performed by incubating the harvested virus supernatant with J76 cells. The cells were expanded and tetramer staining of the TCR-transduced J76 cells was performed 5 days post infection to validate the specificity of the overexpressed TCRs.

### CD69 upregulation

T2 HLA-A2 expressing antigen-presenting cells (CRL-1992, ATCC) were maintained in RPMI containing 10% FBS, HEPES and L-glutamine. T2 cells were pulsed with the desired peptide concentration overnight at 37°C. The cells were then washed to remove excess peptide. The peptide-pulsed T2 cells were co-cultured with TCR-transduced J76 cells for 90 minutes. Cells were harvested and stained with anti-CD8, anti-CD3 and anti-CD69 for 30 minutes in staining buffer. Cells were washed twice and analyzed via flow cytometry using a Calibur (BD Biosciences). Assay was performed in duplicate.

### Soluble HLA-A2/YVL-BR protein production and crystallization

Soluble HLA-A2/YVL-BR complexes were prepared by folding urea-solubilized bacterially-expressed inclusion bodies of HLA-A2 heavy chain and human βeta2-microglobulin in the presence of 5mg/L synthetic YVL-BR peptide essentially as described [55], followed by concentration and buffer exchange into 10mM Tris-Cl (pH 8.0) using a tangential flow concentrator. Folded HLA-A2/peptide complexes were isolated from the buffer-exchanged folding mixture by a series of chromatography steps consisting of Hitrap Q and Mono Q ion exchange and S-200 gel-filtration columns (GE healthcare). Crystals were grown from purified HLA-A2/YVL-BR by sitting drop vapor diffusion using 10.5% (w/v) PEG 4000, 35 mM Tris base/ HCl (pH 8.5), 70 mM Li_2_SO_4_. Crystals were briefly soaked in 1:1 mixture of saturated sucrose and reservoir buffer for cryoprotection and flash-frozen in liquid nitrogen and sent to LRL-CAT beamline at the Advanced Photon Source (Argonne, IL USA).

### HLA-A2/YVL-BR Structure determination and refinement

Diffraction data extending to ~ 3.2Å collected from a single crystal were integrated and indexed using Mosflm [56]. Initially data were indexed in a C2 unit cell (189.2 x 49.7 x 291.6 Å, β = 94.5°), with molecular replacement using Phaser [57] identifying four copies per asymmetric unit of a HLA-A2 model [26] with TFZ = 22. However, refinement of this model stalled at Rfree = 0.42. Re-examination of the diffraction pattern identified a lattice of weak spots spaced between the stronger spots originally indexed (S4A Fig), and the data were reindexed in a P2_1_ unit cell (189.9 x 100.2 x 292.4 Å, β = 94.4°). The newly identified spots comprise the k = 2n+1 and h+(k/2) = 2n+1 sets (S4 Fig). Additional molecular replacement, symmetry considerations, and examination of composite-omit maps calculated using CCP4i [58] identified 20 copies of HLA-A2 per asymmetric unit (S4B Fig). The molecules are arranged in two layers viewed looking into the ac plane (S4C Fig); slight differences can be observed between these layers, and between similarly oriented molecules within the same layer (S4D Fig). These differences, along with four molecules (A,J,K,T) not identified in the C2 cell, explain the lower symmetry and strong translational pseudosymmetry that resulted in weak intensities for the k = 2n+1 and h+(k/2) = 2n+1 spots. After identification of the correct crystallographic and non-crystallographic symmetries, clear electron density covering all peptide atoms was observed in 20-fold averaged composite omit maps (S4E Fig), and a model for the YVL-BR peptide was built using Coot [59]. Refinement using Phenix [60] proceeded smoothly despite the relatively low resolution when dihedral restraints to a higher-resolution reference model were provided. Models for the YVL-BR peptide, which was not included in the reference model restraints, did not vary significantly between the non-crystallographically related copies (S4F Fig). A paired refinement test [61] confirms 3.3 Å as a suitable resolution cutoff. Final refinement statistics, shown in S3 Table are within the range of other structures determined at this resolution in the Protein Data Bank. PyMOL (The PyMOL Molecular Graphics System, Version 2.0 Schrodinger, LLC) was used for graphical representation of molecular structures.

## Supporting information

S1 Fig Flow cytometry analysis of J76 cells transduced with WT or mutant K113A TCRs and GFP, as a mock control, and stained with the cognate A2-YVL-BR (top) or an irrelevant A2-GLC-BM (bottom) tetramer and CD3 antibody. The CDR3α and β amino acid sequences of the TCRs are shown. Bold: residue that has been mutated.
(PDF)

S2 Fig **The KD motif is partially non-germline and is encoded by different nucleotide sequences (A & B).** aa: amino acid; nt: nucleotide; bold: N nucleotide additions.
(PDF)

S3 Fig Hierarchical clustering of GLC-BM-specific TCRα/β in AIM and CONV. TCRαβ clustering along with corresponding TCR logos for GL-BM-specific CD8 T-cell responses in AIM and CONV. Number on the branches and next to TCR logos depicts number of TCRs contributing to the cluster. Color of the branches indicates the TCR probability generation scores. The bar at the bottom of the CDR3 logo is color-coded by the source of the nucleotide. Light grey, red, black, and dark grey denote that the nucleotides encoding those amino acid residues originate from the V, N, D and J regions, respectively. Analyses are based on Dash *et al*. [20].
(PDF)

S4 Fig HLA-A2/YVL-BR structure determination and refinement. **(A)** *Top*, Representation of the (h,k,0) plane, showing weak intensities in the k = 2n+1 layers (k has odd values, alternating horizontal rows) and the h+k/2 = 2n+1 layers (h has odd values in the lines where k is even, alternating spots within the stronger horizontal rows). *Bottom*, average mean spot intensities for various sets of diffraction spots. **(B)** Views of the P2_1_ unit cell and asymmetric unit with HLA-A2/YVL-BR molecules shown in different colored CPK models. **(C)** Ribbon diagram showing orientation of HLA-A2/YVL-BR molecules within the asymmetric unit. **(D)** Small rotational differences between some of the non-crystallographically related molecules break the apparent C2 symmetry responsible for the strong set of diffraction spots, resulting in the observed translational pseudosymmetry. **(E)** Composite omit 2Fo-Fc electron density in the vicinity of the VYL-BR peptide (blue bonds), with HLA-A2 shown in ribbon representation. **(F)** Overlay of 20 HLA-A2/YVL-BR models.
(PDF)

S1 Table Characteristics of study population. ^1^Single-cell paired TCRαβ sequencing was performed on tetramer sorted CD8 T cells of all four donors at presentation with AIM and 5–8 months later. ^2^Time elapsed between AIM and CONV. ^3^Frequency of HLA-A2 restricted GLC or YVL tetramer+ cells within CD3+ CD8+ T cells in PBMCs isolated from each respective donor. ^4^B cells were not available from this donor to perform a viral load assay. AIM: acute infectious mononucleosis; CONV: convalescence; M: male; F: Female.
(DOCX)

S2 Table Paired single-cell GLC-specific TCR amino acid sequences. ^**1**^Color-coded by CDR3α clones. ^2^Color-coded by CDR3β clones.
(DOCX)

S3 Table HLA-A2 / YVL data collection and refinement statistics. (DOCX)

S4 Table Paired single-cell epitope-specific TCR nucleotide sequences. (XLSX)
