# Supplementary figures and images for: CDR3α drives selection of the immunodominant Epstein Barr virus (EBV) BRLF1-specific CD8 T cell receptor repertoire in primary infection
Source: PLoS Pathog. 2019 Nov 25;15(11):e1008122. doi: 10.1371/journal.ppat.1008122 (PMC6901265; doi:10.1371/journal.ppat.1008122)

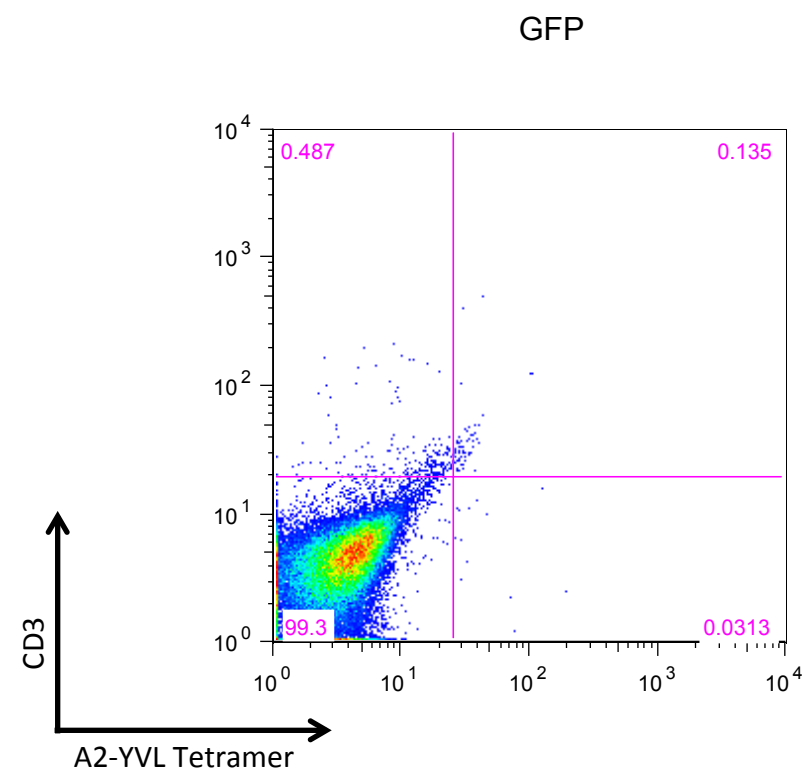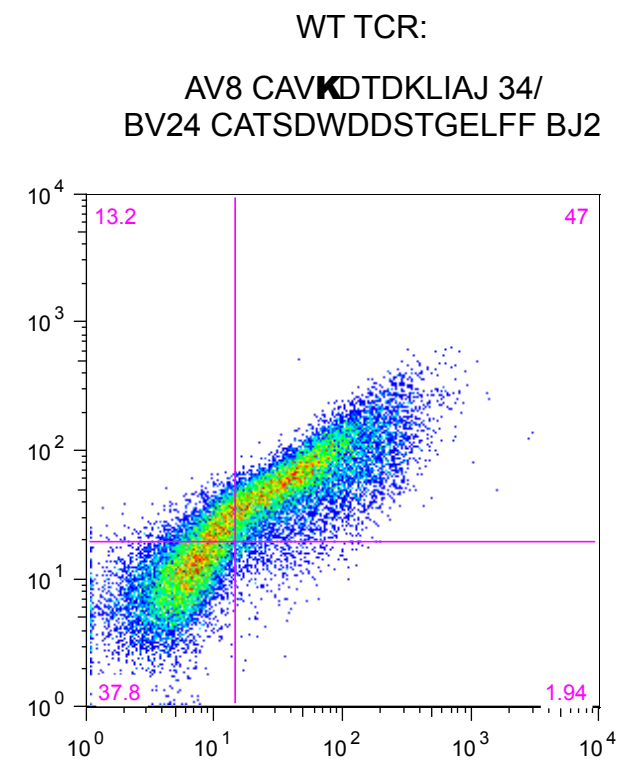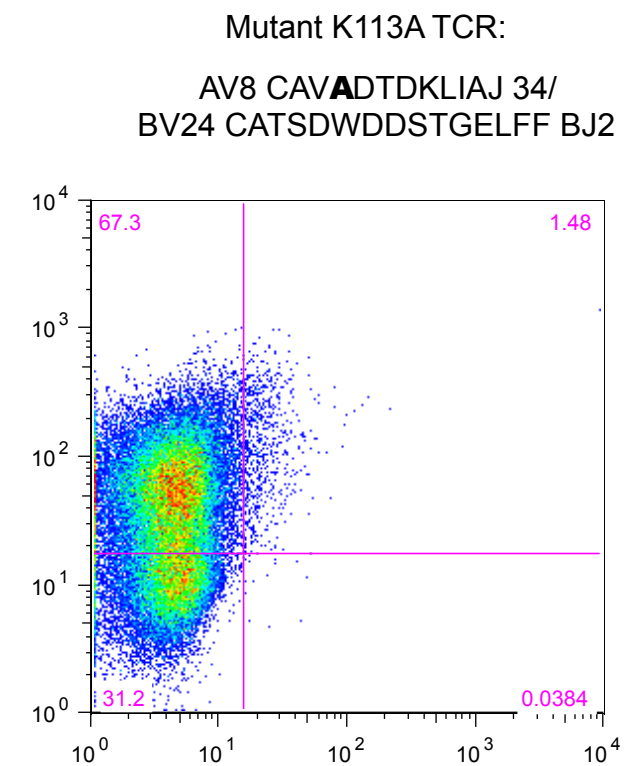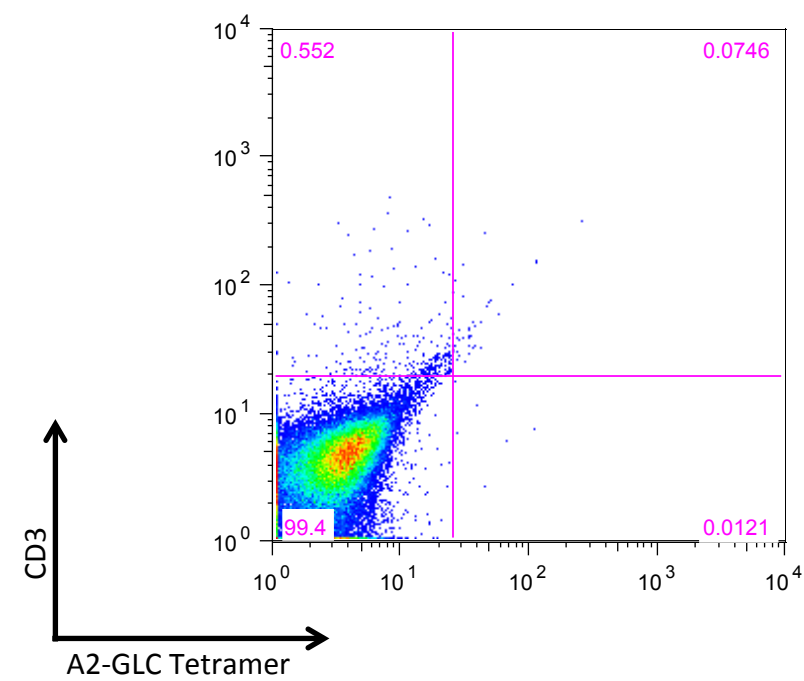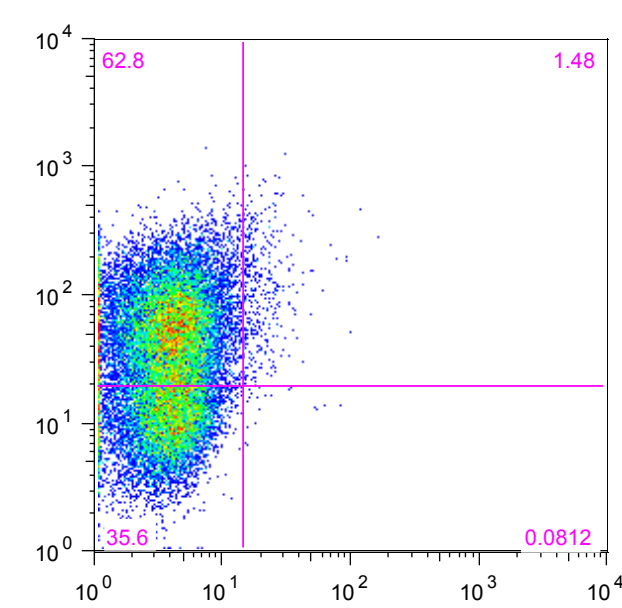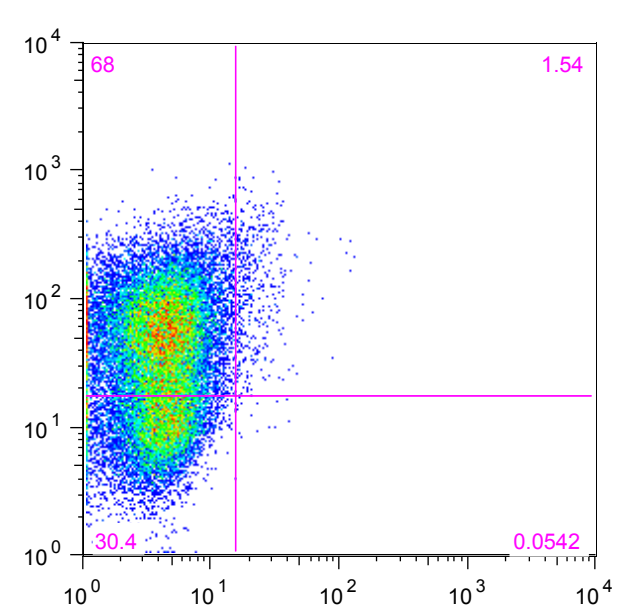

Supplement: S1 Fig — The CDR3α and β amino acid sequences of the TCRs are shown. Bold: residue that has been mutated. (PDF) [file ppat.1008122.s001.pdf]

**A**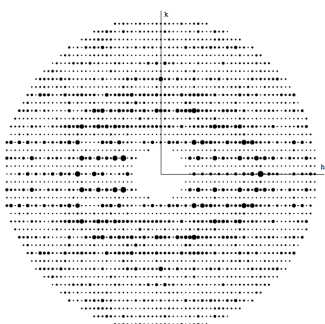**B****Asymmetric unit**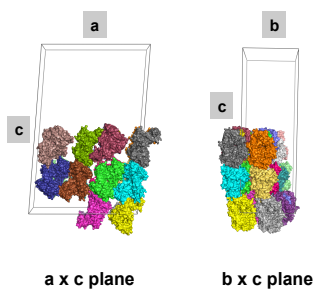**Unit cell**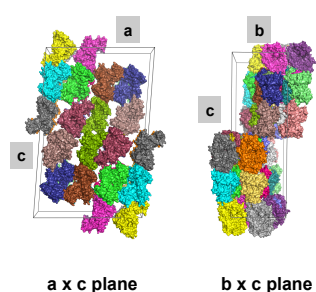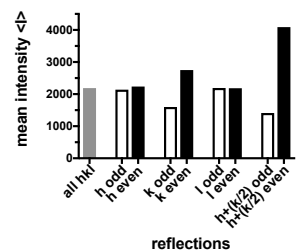**C**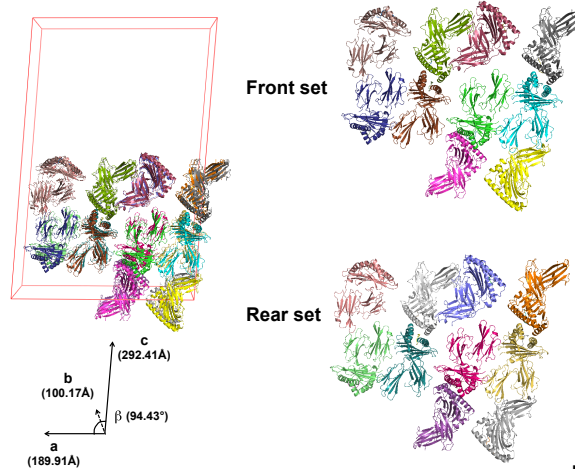**E**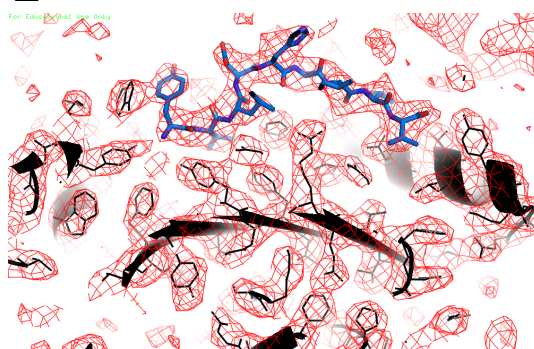**D**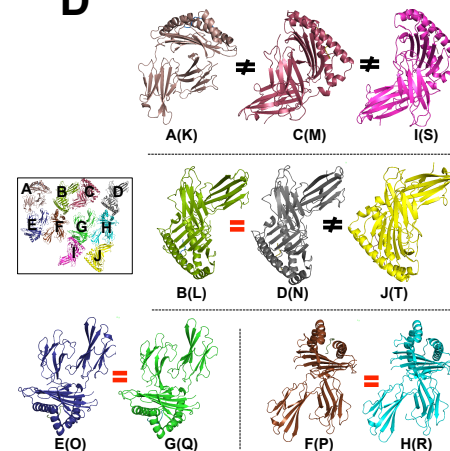**F**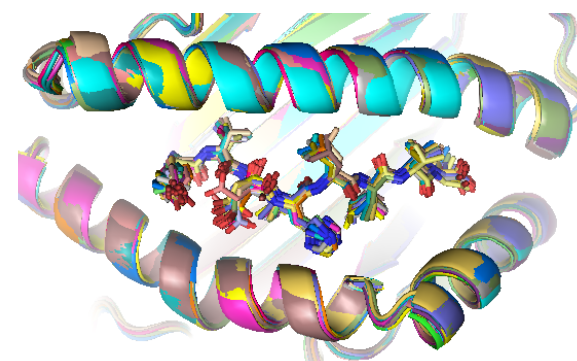

Supplement: S4 Fig — (A) Top, Representation of the (h,k,0) plane, showing weak intensities in the k = 2n+1 layers (k has odd values, alternating horizontal rows) and the h+k/2 = 2n+1 layers (h has odd values in the lines where k is even, alternating spots within the stronger horizontal rows). Bottom, average mean spot intensities for various sets of diffraction spots. (B) Views of the P21 unit cell and asymmetric unit with HLA-A2/YVL-BR molecules shown in different colored CPK models. (C) Ribbon diagram showing orientation of HLA-A2/YVL-BR molecules within the asymmetric unit. (D) Small rotational differences between some of the non-crystallographically related molecules break the apparent C2 symmetry responsible for the strong set of diffraction spots, resulting in the observed translational pseudosymmetry. (E) Composite omit 2Fo-Fc electron density in the vicinity of the VYL-BR peptide (blue bonds), with HLA-A2 shown in ribbon representation. (F) Overlay of 20 HLA-A2/YVL-BR models. (PDF) [file ppat.1008122.s004.pdf]
