# Supplementary material for: CDR3α drives selection of the immunodominant Epstein Barr virus (EBV) BRLF1-specific CD8 T cell receptor repertoire in primary infection
Source: PLoS Pathog. 2019 Nov 25;15(11):e1008122. doi: 10.1371/journal.ppat.1008122 (PMC6901265; doi:10.1371/journal.ppat.1008122)
Supplement: S2 Fig — The KD motif is partially non-germline and is encoded by different nucleotide sequences (A & B). aa: amino acid; nt: nucleotide; bold: N nucleotide additions. (PDF) [file ppat.1008122.s002.pdf]

A

CDR3a aa →	C	A	V	**K**	**D**	T	D	K	L	I	F
CDR3a nt →	tgt	gcc	gtg	aa**g**	**g**ac	acc	gac	aag	ctc	atc	ttt
	AV8.1				AJ34						

B

CDR3a aa →	C	A	V	**K**	**D**	T	D	K	L	I	F
CDR3a nt →	tgt	gcc	gtg	aa**a**	**g**ac	acc	gac	aag	ctc	atc	ttt
	AV8.1				AJ34						
