# Supplementary material for: CDR3α drives selection of the immunodominant Epstein Barr virus (EBV) BRLF1-specific CD8 T cell receptor repertoire in primary infection
Source: PLoS Pathog. 2019 Nov 25;15(11):e1008122. doi: 10.1371/journal.ppat.1008122 (PMC6901265; doi:10.1371/journal.ppat.1008122)
Supplement: S3 Fig — TCRαβ clustering along with corresponding TCR logos for GL-BM-specific CD8 T-cell responses in AIM and CONV. Number on the branches and next to TCR logos depicts number of TCRs contributing to the cluster. Color of the branches indicates the TCR probability generation scores. The bar at the bottom of the CDR3 logo is color-coded by the source of the nucleotide. Light grey, red, black, and dark grey denote that the nucleotides encoding those amino acid residues originate from the V, N, D and J regions, respectively. Analyses are based on Dash et al. [20]. (PDF) [file ppat.1008122.s003.pdf]

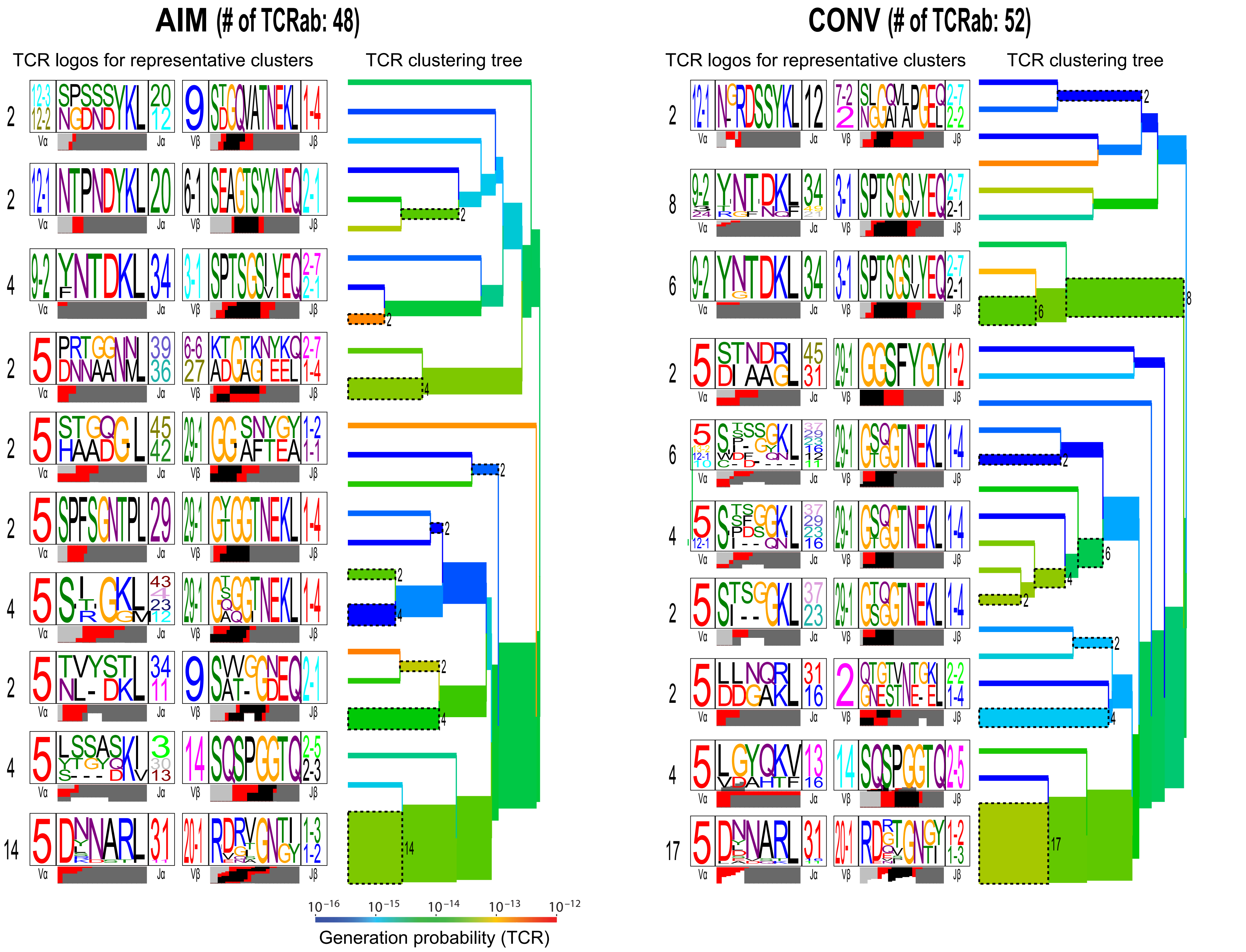
AIM (# of TCRab: 48)
TCR logos for representative clusters
TCR clustering tree
CONV (# of TCRab: 52)
TCR logos for representative clusters
TCR clustering tree
Vα
Jα
Vβ
Jβ
$10^{-16}$
$10^{-15}$
$10^{-14}$
$10^{-13}$
$10^{-12}$
Generation probability (TCR)
