# Supplementary material for: CDR3α drives selection of the immunodominant Epstein Barr virus (EBV) BRLF1-specific CD8 T cell receptor repertoire in primary infection
Source: PLoS Pathog. 2019 Nov 25;15(11):e1008122. doi: 10.1371/journal.ppat.1008122 (PMC6901265; doi:10.1371/journal.ppat.1008122)
Supplement: S1 Table — 1Single-cell paired TCRαβ sequencing was performed on tetramer sorted CD8 T cells of all four donors at presentation with AIM and 5–8 months later. 2Time elapsed between AIM and CONV. 3Frequency of HLA-A2 restricted GLC or YVL tetramer+ cells within CD3+ CD8+ T cells in PBMCs isolated from each respective donor. 4B cells were not available from this donor to perform a viral load assay. AIM: acute infectious mononucleosis; CONV: convalescence; M: male; F: Female. (DOCX) [file ppat.1008122.s005.docx]

**S1 Table. Characteristics of study population.**

| Donor ID^1^ | Gender | Time point | Time (months)^2^ | EBV viral load (log_10_ copies/million B cells) | GLC^3^ (%) | YVL^3^ (%) |
| --- | --- | --- | --- | --- | --- | --- |
| E1603 | M | AIM | 0 | 2.03 | 0.6 | 2.9 |
|  |  | CONV | 6 | 1.91 | 0.5 | 0.7 |
| E1632 | F | AIM | 0 | 4.36 | 1.1 | 2 |
|  |  | CONV | 7 | No data^4^ | 0.2 | 0.1 |
| E1655 | F | AIM | 0 | 5.05 | 1.6 | 1.3 |
|  |  | CONV | 5 | 3.3 | 0.2 | 0.1 |
| E1651 | F | AIM | 0 | 4.14 | 1.7 | 4.5 |
|  |  | CONV | 8 | Not detected | 0.1 | 0.1 |
