# Supplementary material for: CDR3α drives selection of the immunodominant Epstein Barr virus (EBV) BRLF1-specific CD8 T cell receptor repertoire in primary infection
Source: PLoS Pathog. 2019 Nov 25;15(11):e1008122. doi: 10.1371/journal.ppat.1008122 (PMC6901265; doi:10.1371/journal.ppat.1008122)
Supplement: S2 Table — 1Color-coded by CDR3α clones. 2Color-coded by CDR3β clones. (DOCX) [file ppat.1008122.s006.docx]

| **S2 Table. Paired single-cell GLC-specific TCR amino acid sequences.** | | | | |
| --- | --- | --- | --- | --- |
|  |  |  | Count | Count |
| **Donor ID** | **CDR3α^1^** | **CDR3β^2^** | **AIM** | **CONV** |
| E1603 | AV5CAEDNNARLMF AJ31 | BV20CSARDGTGNGYTF BJ1 |  | 3 |
| E1603 | AV5CAEDNNARLMF AJ31 | BV20CSARDQTGNGYTF BJ1 |  | 4 |
| E1603 | AV5CAELDVQKLVS AJ16 | BV20CSARDRVGNTIYF BJ1 |  | 1 |
| E1603 | AV5CAEDYNARLMF AJ31 | BV20CSARDRVGNTIYF BJ1 |  | 2 |
| E1603 | AV5CAEDKNARLMF AJ31 | BV20CSARDRVGNTIYF BJ1 | 1 |  |
| E1603 | AV20CAWQGNYGQNFVF AJ26 | BV20CSARDRVGNTIYF BJ1 | 2 |  |
| E1603 | AV20CARNGYNDYKLSF AJ20 | BV20CSARDRVGNTIYF BJ1 | 1 |  |
| E1603 | AV5CAEDENARLMF AJ31 | BV20CSARDVPGNTIYF BJ1 |  | 1 |
| E1603 | AV5CAESGRGKLIF AJ12 | BV29CSVGAGGTNEKLFF BJ1 | 1 |  |
| E1603 | AV26CILTGGGNKLTF AJ10 | BV29CSVSPVDYYTQYF BJ2 |  | 1 |
| E1603 | AV5CAEVDAHTFLF AJ16 | BV2CASQNGTVNTGELFF BJ2 | 1 |  |
| E1603 | AV5CAELDGQKLLF AJ16 | BV2CASQNGTVNTGELFF BJ2 |  | 1 |
| E1603 | AV20CARKGNNGYKLVF AJ26 | BV2CASSEGQIAPGELFF BJ2 | 1 |  |
| E1603 | AV12SAMSGYTDYKLSF AJ20 | BV2CASSEGQIAPGELFF BJ2 | 1 |  |
| E1603 | AV12CVVNGEDSSYKLIF AJ12 | BV2CASSEGQIAPGELFF BJ2 | 3 |  |
| E1603 | AV9CAGYNTDKLIF AJ34 | BV3CASSPTSGSVYEQFF BJ2 |  | 1 |
| E1603 | AV10CVVSEGKLIF AJ34 | BV6CASELWTGHNEQFF BJ2 |  | 1 |
| E1603 | AV12CAMSGSNDYKLSF AJ20 | BV9CASSDGQVATNEKLFF BJ1 | 5 | 5 |
| E1632 | AV9CASYGTDKLIF AJ34 | BV19CCRSPRLARRRIYEQYF BJ2 |  | 1 |
| E1632 | AV5CAEDNNARLMF AJ31 | BV20CSARDQTGNGYTF BJ1 |  | 1 |
| E1632 | AV5CAEDLNARLMF AJ31 | BV20CSARDRIGNTIYF BJ1 | 2 | 4 |
| E1632 | AV5CAEDNNARLMF AJ31 | BV20CSARDRTGNGYTF BJ1 | 1 |  |
| E1632 | AV5CAEDRDSTLTF AJ11 | BV20CSARVGAGNTIYF BJ1 | 1 |  |
| E1632 | AV5CAEHAGQGNLIF AJ42 | BV29CSVGGQANTEAFF BJ2 |  | 1 |
| E1632 | AV5CAEHAGQGNLIF AJ42 | BV29CSVGGQANTEAFF BJ1 |  | 3 |
| E1632 | AV13CATWGF AJ11 | BV29CSVGTGGTNEKLFF BJ1 |  | 1 |
| E1632 | AV9CASYGTDKLIF AJ34 | BV3CASSPTSGSIYEQYF BJ2 |  | 1 |
| E1632 | AV9CALYNTDKLIV AJ34 | BV3CASSPTSGSIYEQYF BJ2 | 1 |  |
| E1632 | AV9CALYNTDKLIF AJ34 | BV3CASSPTSGSIYEQYF BJ2 | 9 | 5 |
| E1632 | AV3CAVRDGGNFNKFYF AJ21 | BV3CASSPTSGSIYEQYF BJ2 |  | 1 |
| E1632 | AV9CALFNTDKLIF AJ34 | BV3CASSPTSGSVYEQFF BJ2 | 1 |  |
| E1632 | AV20WGGNWDGSYKLIF AJ12 | BV7CASNLGQILPGEQYF BJ2 | 1 |  |
| E1632 | AV12CVVNRDSSYKLIF AJ12 | BV7CASNLGQILPGEQYF BJ2 |  | 2 |
| E1651 | AV8CAVVGTGNQFYF AJ49 | BV10CAISDDRGPYEQYF BJ2 | 1 |  |
| E1651 | AV5CAELSDKIIF AJ30 | BV14CARSQSPGGTQYF BJ2 | 1 |  |
| E1651 | AV5CAEAMAGNMLTF AJ39 | BV15CATSITSGSQTQYF BJ2 |  | 1 |
| E1651 | AV5CAVDNNARLMF AJ31 | BV20CSARDETGNGYTF BJ1 |  | 2 |
| E1651 | AV5CAEDADSTLTF AJ11 | BV20CSARDGTGNTIYF BJ1 |  | 1 |
| E1651 | AV5CAEDNNARLMF AJ31 | BV20CSARDQIGNGYTF BJ1 | 1 |  |
| E1651 | AV5CAEDNNARLMF AJ31 | BV20CSARDRTGNGYTF BJ1 |  | 4 |
| E1651 | AV17CALYNTDKLIF AJ34 | BV20CSARDRTGNGYTF BJ1 |  | 1 |
| E1651 | AV12CVVNVPNDYKLSF AJ20 | BV20CSARDRTGNGYTF BJ1 |  | 1 |
| E1651 | AV5CAEDRYSTLTF AJ11 | BV20CSARDSTGNGYTF BJ1 | 1 |  |
| E1651 | AV5WTEDQRGKLMF AJ23 | BV20CSVRDRVGNTIYF BJ1 | 1 |  |
| E1651 | AV23CAVTGGAANKLIF AJ32 | BV27CASSPGTSGYYNEQFF BJ2 |  | 1 |
| E1651 | AV12CAISNFGNEKLTF AJ48 | BV3CASSFTSGSIYEQYF BJ2 | 2 |  |
| E1651 | AV9CALYNTDKLIF AJ34 | BV3CASSPTSGSIYEQFF BJ2 |  | 2 |
| E1651 | AV9CAFYNTDKLIF AJ34 | BV3CASSPTSGSIYEQFF BJ2 | 1 |  |
| E1651 | AV9CALYNTDKLIF AJ34 | BV3CASSPTSGSVYEQFF BJ2 |  | 1 |
| E1651 | AV1VYVSDKDSTDKLIF AJ34 | BV3CASSPTSGSVYEQYF BJ2 | 1 |  |
| E1651 | AV12CVVNTPNDYKLSF AJ20 | BV6CASSEAGLATTMSSSS BJ2 | 1 |  |
| E1651 | AV12RVVNTPGNDKFTF AJ20 | BV6CASSEAGTSYYNEQFF BJ2 | 2 |  |
| E1651 | AV12CVVNTPNDYKLTF AJ20 | BV6CASSEAGTSYYNEQFF BJ2 | 1 |  |
| E1651 | AV12CVVNTPNDYKLSF AJ20 | BV6CASSEAGTSYYNEQFF BJ2 | 11 | 4 |
| E1651 | AV12CVVNRPNDYKLSF AJ20 | BV6CASSESAWVAGGSDTQYF BJ2 |  | 1 |
| E1651 | AV12CVVNMEGYSTLTF AJ11 | BV6CASSESPMWDPRYGYTF BJ1 |  | 1 |
| E1651 | AV4CLVVNDYKLSF AJ20 | BV7CASSLAFSGLLTDTQYF BJ2 | 1 |  |
| E1651 | AV30CGTEILNDYKLSF AJ20 | BV9CASSEGQLSSGNTIYF BJ1 |  | 1 |
| E1655 | AV5CAELGYQKVTF AJ13 | BV14CANSQSPGGTQFF BJ2 |  | 1 |
| E1655 | AV5CAVYSSASKIIF AJ3 | BV14CASSQSPGGTQYF BJ2 | 1 |  |
| E1655 | AV5CAESTSASKIIF AJ3 | BV14CASSQSPGGTQYF BJ2 | 1 |  |
| E1655 | AV5CAELGYQKVTF AJ13 | BV14CASSQSPGGTQYF BJ2 | 3 | 5 |
| E1655 | AV5CADRQSQKDTF AJ13 | BV14CASSQSPGGTQYF BJ2 | 1 |  |
| E1655 | AV5CAVSTSYGKLTF AJ52 | BV20CSAPRAGGGQETQYF BJ2 |  | 1 |
| E1655 | AV5CAEDRMPEIMF AJ31 | BV20CSARDGTGNGYTF BJ1 |  | 1 |
| E1655 | AV5CAEDNNARLMF AJ31 | BV20CSARDGTGNGYTF BJ1 |  | 1 |
| E1655 | AV5CAFDNNARLMF AJ31 | BV20CSARDGVGNGYTF BJ1 | 1 |  |
| E1655 | AV5CAEDLNARLMF AJ31 | BV20CSARFRENSGNTIYF BJ1 | 5 | 7 |
| E1655 | AV5CAEDNNARLMF AJ31 | BV25CASSGARDTQYF BJ2 |  | 1 |
| E1655 | AV5CAEDRTGANNLFF AJ36 | BV27CASADGAKIYEQYF BJ2 | 1 |  |
| E1655 | AV5CPGDINARLMF AJ31 | BV29CSVGGSFYGYTF BJ1 |  | 1 |
| E1655 | AV5CAESTADGLTF AJ45 | BV29CSVGGSFYGYTF BJ1 | 2 | 1 |
| E1655 | AV5CAESIGGMRF AJ43 | BV29CSVGQGGTNEKLFF BJ1 | 1 |  |
| E1655 | AV5CAESPFSGNTPLVF AJ29 | BV29CSVGSGGTNEKLFF BJ1 |  | 3 |
| E1655 | AV5CAESISGGKLIF AJ23 | BV29CSVGSQGINEKLFF BJ1 | 1 |  |
| E1655 | AV5VSESISGRKLIF AJ23 | BV29CSVGSQGTNEKLFF BJ1 | 1 |  |
| E1655 | AV5CAESISGGKLIF AJ23 | BV29CSVGSQGTNEKLFF BJ1 |  | 1 |
| E1655 | AV12CVVSWFSDGQKLLF AJ16 | BV29CSVGSQGTNEKLFF BJ1 |  | 1 |
| E1655 | AV10CVVCRMDSSYKLIF AJ12 | BV29CSVGSQGTNEKLFF BJ1 |  | 1 |
| E1655 | AV5CAESTGKLIF AJ4 | BV29CSVGTGGTNEKLFF BJ1 | 2 |  |
| E1655 | AV5CAESTGKLIF AJ37 | BV29CSVGTGGTNEKLFF BJ1 |  | 3 |
| E1655 | AV5CAESPFSGNTPLVF AJ29 | BV29CSVGTGGTNEKLFF BJ1 | 1 |  |
| E1655 | AV5CAEDLNARLMF AJ31 | BV2CAVGTESTNEKLFF BJ1 |  | 1 |
| E1655 | AV24CAFTNTGNQFYF AJ49 | BV3CASSPTSGSVYEQYF BJ2 |  | 1 |
| E1655 | AV13CAASQIGNEKLTF AJ48 | BV5CASSPWDRGATNEKLFF BJ1 | 1 |  |
| E1655 | AV5CAEPNNAGNMLTF AJ39 | BV6CASKTGTGNEKLFF BJ1 | 1 |  |
| E1655 | AV5CSESNKDGNYQLIW AJ33 | BV6SSSADLRTRWLLIKPSL BJ1 | 1 |  |
| E1655 | AV5CAENLYSTLTF AJ11 | BV9CASSATGGDEQFF BJ2 | 1 |  |
| E1655 | AV12CAMGGSNDYKLSF AJ20 | BV9CASSGNPQTGPMNTEAFF BJ1 | 1 |  |
| E1655 | AV5GTGTDDYKLSF AJ20 | BV9CASSPGLVSSGELFF BJ2 | 1 |  |
| E1655 | AV30CGTEIPHDYKLSF AJ20 | BV9CASSTGQLSSGNTIYF BJ1 | 3 |  |
| E1655 | AV12CAVNPDSSYKLIF AJ12 | BV9CASSTGQVATNEKLFF BJ1 | 1 |  |
| E1655 | AV5CAETVDKLIF AJ34 | BV9CASSVVGNEQFF BJ2 | 1 |  |
