# Supplementary material for: CDR3α drives selection of the immunodominant Epstein Barr virus (EBV) BRLF1-specific CD8 T cell receptor repertoire in primary infection
Source: PLoS Pathog. 2019 Nov 25;15(11):e1008122. doi: 10.1371/journal.ppat.1008122 (PMC6901265; doi:10.1371/journal.ppat.1008122)
Supplement: S3 Table — (DOCX) [file ppat.1008122.s007.docx]

**S3 Table. HLA-A2 / YVL data collection and refinement statistics.**

Wavelength (Å) 0.97931

Resolution range (Å) 112 - 3.3 (3.4 - 3.3)

Space group P 1 21 1

Unit cell (Å, °) 189.91 100.17 292.41 90 94.43 90

Total reflections 318439 (30941)

Unique reflections 164058 (16130)

Multiplicity 1.9 (1.9)

Completeness (%) 0.99 (0.98)

Mean I/sigma(I) 3.08 (1.37)

Wilson B-factor 47.80

R-merge 0.2073 (0.5542)

R-meas 0.2932 (0.7838)

CC1/2 0.952 (0.548)

CC* 0.988 (0.842)

Reflections used in refinement 164058 (16109)

Reflections used for R-free 8301 (816)

R-work 0.254 (0.291)

R-free 0.306 (0.301)

CC(work) 0.925 (0.759)

CC(free) 0.928 (0.757)

Number of non-hydrogen atoms 63194

Protein residues 7680

RMS(bonds) 0.011

RMS(angles) 1.50
